# Adaptation in Young Military Recruits: Protocol for the Advancing Research on Mechanisms of Resilience (ARMOR) Prospective Longitudinal Study

**DOI:** 10.2196/51235

**Published:** 2023-10-04

**Authors:** Melissa A Polusny, Craig A Marquardt, Michelle Hubbling, Emily Hagel Campbell, Paul A Arbisi, Nicholas D Davenport, Kelvin O Lim, Shmuel Lissek, Jonathan D Schaefer, Scott R Sponheim, Ann S Masten, Siamak Noorbaloochi

**Affiliations:** 1 Minneapolis Veterans Affairs Health Care System Minneapolis, MN United States; 2 Center for Care Delivery and Outcomes Research Minneapolis, MN United States; 3 Department of Psychiatry and Behavioral Sciences University of Minnesota Medical School Minneapolis, MN United States; 4 Department of Psychology University of Minnesota-Twin Cities Minneapolis, MN United States; 5 Department of Psychology Vanderbilt University Nashville, TN United States; 6 Institute of Child Development University of Minnesota-Twin Cities Minneapolis, MN United States; 7 Department of Medicine University of Minnesota Medical School Minneapolis, MN United States

**Keywords:** study protocol, military personnel, longitudinal studies, resilience, adaptive behavior, stress, adversity, mechanisms, protective factors

## Abstract

**Background:**

Military services provide a unique opportunity for studying resilience, a dynamic process of successful adaptation (ie, doing well in terms of functioning and symptoms) in response to significant adversity. Despite the tremendous interest in positive adaptation among military service members, little is known about the processes underlying their resilience. Understanding the neurobiological, cognitive, and social mechanisms underlying adaptive functioning following military stressor exposure is essential for enhancing the resilience of military service members.

**Objective:**

The primary objective of the Advancing Research on Mechanisms of Resilience (ARMOR) longitudinal study is to characterize the trajectories of positive adaptation among young military recruits in response to basic combat training (BCT), a well-defined, uniform, and 10-week period of intense stress (aim 1), and identify promotive and protective processes contributing to individual variations in resilience (aim 2). The secondary objective is to investigate the pathways by which neurobehavioral markers of self-regulation assessed using electroencephalography and magnetic resonance imaging contribute to adaptive trajectories (aim 3).

**Methods:**

ARMOR is an ongoing, prospective longitudinal cohort study of young military recruits who recently joined the National Guard but have not yet shipped out for BCT. Participants (N=1201) are assessed at 5 time points over the initial >2 years of military service beginning before BCT (baseline) and followed up at 2 weeks and 6, 12, and 18 months after BCT. Participants complete web-based questionnaires assessing vulnerability and protective factors, mental health, and socioemotional functioning at each time point and a battery of neurocognitive tests at time 0. A subset of participants also complete structured diagnostic interviews and additional self-report measures and perform neurobehavioral tasks before and after BCT during electroencephalography sessions and before BCT only during magnetic resonance imaging sessions.

**Results:**

This UG3/UH3 project was initially funded in August 2017, with the UG3 pilot work completed at the end of 2018. The UH3 phase of the project was funded in March 2019. Study enrollment for the UH3 phase began on April 14, 2019, and ended on October 16, 2021. A total of 1201 participants are enrolled in the study. Follow-up data collection for the UH3 phase is ongoing and projected to continue through February 2024. We will disseminate the findings through conferences, webinars, open access publications, and communications with participants and stakeholders.

**Conclusions:**

The ARMOR study provides a rich data set to identify the predictors and mechanisms of resilient and nonresilient outcomes in the context of military stressors, which are intended to empirically inform the development of prevention and intervention strategies to enhance the resilience of military trainees and potentially other young people facing significant life challenges.

**International Registered Report Identifier (IRRID):**

DERR1-10.2196/51235

## Introduction

### Background

Across the military career life cycle, service members are at a considerable risk for exposure to stressors that may impact their health, well-being, and performance [1-3]. Extensive research has identified factors that contribute to the risk for psychopathology (eg, posttraumatic stress symptoms and depression) following combat exposure and other military-related stressors [4-6]. However, mounting evidence suggests that most individuals show resilience, adapting successfully to risk and adversity [7,8]. Understanding the neurobiological, cognitive, and social mechanisms underlying successful adaptation following military stressor exposure is essential for designing prevention and intervention strategies to enhance resilience in military populations [9]. However, the mechanisms and processes that facilitate resilience following military stressors remain poorly understood.

Most studies on resilience within the military context have operationalized resilience as a static, trait-like attribute or have relied on cross-sectional designs [10]. One-time assessments of adaptation, adversity exposure, and resilience, particularly through self-report questionnaires, provide very limited insights into risk and protective processes and their influence on later mental health outcomes. There is now a growing consensus that resilience is a multidimensional and dynamic process that unfolds over time in response to a challenge [11-14]. We define resilience as the capability of a system to adapt successfully through multiple processes to challenges that threaten the system function [12]. In addition, the resilience of an individual draws on support from systems beyond the individual, including supportive relationships, such as battle buddies or a supportive unit [15,16]. Variations in the nature and timing of adversity exposure also influence how individuals adapt, and it is ideal to assess adaptive functioning before, during, and following exposure to well-described uniform stressors. Basic combat training (BCT) provides a systematic and relatively uniform challenge with known timing, so assessments of adversity, potential vulnerabilities, and protective influences, as well as adaptive functioning, can be performed before, during, and following a well-described period of challenge. Therefore, our approach to operationalizing resilience calls for a prospective, longitudinal study that tracks the adaptation of military service members over time with assessments before and after BCT and related, well-defined challenges.

A growing body of longitudinal research with military populations has identified distinct latent classes (groupings) of service members demonstrating similar trajectories of adaptation over time [7,8,17]. This literature suggests that there is significant heterogeneity in people’s responses to comparable stressors. However, these studies have generally examined trajectories following deployment and across the transition from military to civilian life [18,19]. Little research has focused on young military recruits from the outset of their military careers. During the initial years of military service, recruits are faced with numerous challenges (eg, moving away from home, separation from family and friends, dramatic changes in the living environment, and intense military training) as they are encultured into military life. Moreover, for most recruits, this transition from civilian to military life occurs within the context of the transition from adolescence to emerging adulthood [20]. Identifying potential contributing factors linked to risk and resilience early in military service may be particularly useful for effectively intervening in service members during this key developmental transition. Longitudinal studies have also examined a limited number of outcomes in isolation (eg, posttraumatic stress disorder [PTSD], depression, and alcohol use). Our conceptualization of resilience as a multidimensional process in which a person may show successful adaptation within some domains but not others [21] calls for research that assesses multiple outcome domains (eg, internalizing symptoms, externalizing problems, and social-occupational functioning) over time. Finally, prior research has investigated the role of demographic characteristics (eg, age and gender), psychological factors (eg, neuroticism and self-efficacy), and environmental factors (eg, social support and subsequent life stressors) in differentiating individuals who manifest resilience from those who exhibit maladaptive trajectories [8,18,19]. Few studies have integrated neurobehavioral paradigms into longitudinal cohort studies [7,22], so the neurobiological, cognitive, and behavioral processes contributing to trajectory membership remain largely unknown.

Drawing on the aforementioned literature, the Advancing Research on Mechanisms of Resilience (ARMOR) study was established at the Minneapolis Veterans Affairs Health Care System (MVAHCS) and the University of Minnesota-Twin Cities (UMN) [23]. The overarching goal of this prospective, longitudinal cohort study with an embedded laboratory substudy is to develop a comprehensive, multilevel model of resilience to guide the development of prevention and intervention strategies for military trainees and potentially other young people facing significant life challenges.

**Figure 1:**
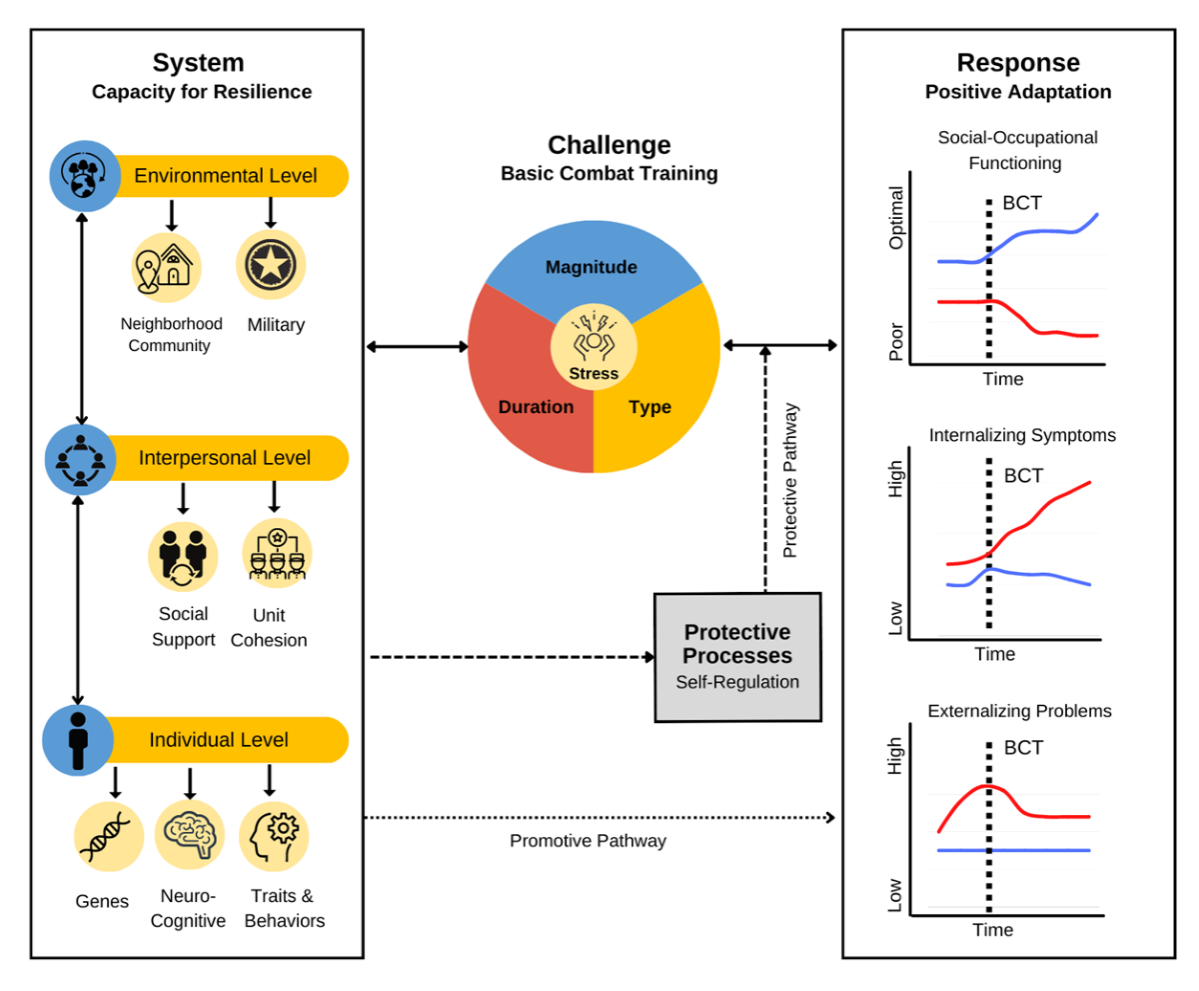
Integrated multilevel model of resilience for military service members. Conceptual model of resilience as a dynamic process in response to the challenges of basic combat training (BCT). Risk and protective factors across multiple levels (individual, social, and environmental) are depicted in the box on the left. Promotive and protective pathways are depicted with dashed and dotted lines, respectively. Positive (blue) and negative (red) responses to the challenges of BCT are shown in the graphs in the box on the right. This figure is from the study by Polusny and Erbes [24] and used under Creative Commons CC-BY) license.

Figure 1 presents the basic conceptual model guiding this study [24]. It recognizes that individuals are embedded within a complex system of risk and protective factors that span multiple levels, from individual neurobiological and behavioral factors to social and broader environmental factors. This multisystemic perspective acknowledges the dynamic and reciprocal relationships among these influences and their joint impact on resilience [25]. The model also emphasizes potential pathways to risk and resilience in the face of adversity (shown as blue and red lines in response to BCT). Challenging life experiences in the past and at present, including childhood adversity or trauma and exposure to military training, can heighten the risk of developing psychopathology. As illustrated by the dotted line in Figure 1, promotive factors, such as cognitive ability and social support, directly contribute to positive adaptation, regardless of the risk level. The dashed line in Figure 1 illustrates how protective factors can act as buffers against the detrimental effects of adversity. In the ARMOR study, the focus is on investigating self-regulation as a key protective process contributing to resilience.

Self-regulation comprises 3 distinct but interrelated neurocognitive processes involving affect, behavior, and cognition, which together can facilitate adaptive responding to adversity [26]. Attentional control involves the ability to concentrate and sustain attention despite distractions, inhibitory control refers to the regulation of maladaptive behavior in favor of goal-directed actions, and behavioral flexibility enables the adaptation of behavioral strategies to meet environmental demands. Previous studies using laboratory-based paradigms have identified significant associations among these self-regulatory processes and measures of trait resilience, implying their potential as stress-buffering mechanisms [27]. However, few studies have examined neurobiological, cognitive, and behavioral processes in relation to resilience trajectory membership [7].

### Objectives

The primary objective of the ARMOR study is to characterize the trajectories of adaptation among young military recruits in response to BCT over the first 2 years of military service (aim 1) and identify promotive factors and protective processes contributing to individual variations in adaptation (aim 2). The secondary objective is to investigate whether neurobehavioral markers of self-regulation are predictive of resilient and nonresilient trajectories (aim 3).

## Methods

### Study Design

**Figure 2:**
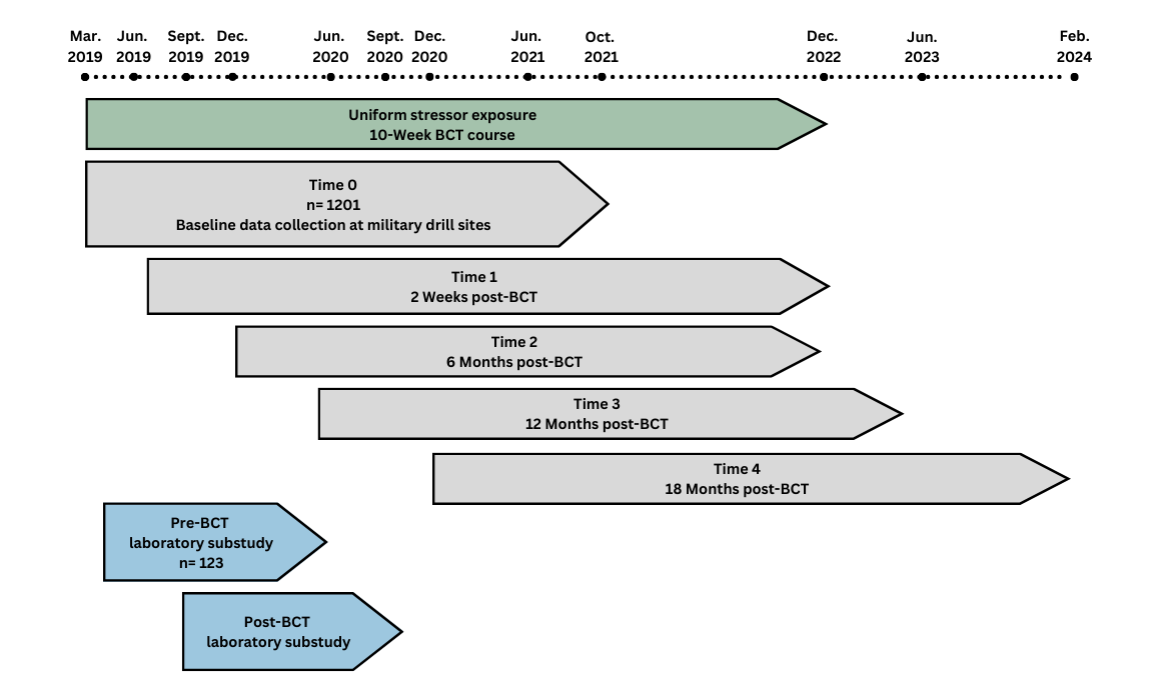
Advancing Research on Mechanisms of Resilience (ARMOR) study timeline. BCT: basic combat training.

ARMOR is a prospective, longitudinal cohort study of young military recruits, including those aged 17 years at study entry, that investigates the mechanisms contributing to manifested resilience across multiple levels of analysis (neural, cognitive, behavioral, and social). The study includes the following two components: (1) a web-based longitudinal survey component, including self-report measures presented via Qualtrics (Qualtrics International Inc) and neurocognitive tests administered using the Penn Computerized Neurocognitive Battery (PCNB); and (2) a laboratory substudy component consisting of clinical diagnostic interviews, additional self-report questionnaires, and a series of performance-based tasks involving electroencephalography (EEG) and magnetic resonance imaging (MRI) assessments. We aimed to enroll a cohort of at least 1200 young military recruits who recently enlisted in the Minnesota Army National Guard. Participant recruitment began on April 14, 2019, and ended on October 16, 2021. Data collection is ongoing and is expected to continue into 2024. As shown in Figure 2, participants were assessed at baseline (time 0) before exposure to a uniform military challenge (BCT) and are currently being followed up at 4 time points (study is ongoing): 2 weeks following return from BCT (time 1) and at 6 months (time 2), 12 months (time 3), and 18 months (time 4) after BCT. A subset of participants from the longitudinal cohort (123/1201, 10.24%) completed the laboratory substudy procedures before shipping out for BCT (before BCT) and after returning from the training (after BCT).

### Context and Setting

This study aims to investigate mental health and adaptive functioning among young military recruits before and immediately after BCT and beyond, offering a unique opportunity to study resilience in response to a well-defined, naturally occurring, and uniform military stressor. BCT is a mandatory, intensive, and 10-week training course that all military recruits must successfully complete. The training environment is highly structured and characterized by low personal autonomy, with strict discipline enforced by drill instructors [28]. One distinctive aspect of BCT is the deliberate introduction of numerous stressors into the training environment, designed to prepare recruits for the challenges they will face in future military life [29]. These challenges include prolonged separation from family and friends, dramatic changes in the living environment, extreme physical demands, mental challenges, and simulated combat. This process is believed to foster unit cohesion and cultivate a strong sense of mental and physical toughness known as the *warrior ethos*. Previous research has shown that BCT has positive effects on cognitive performance, mood, and physical fitness [30-34], and it is associated with increased unit cohesion over time, which, in turn, leads to reduced psychological distress and improvements in tolerating training stressors [35].

Although most military recruits successfully adapt to the challenges of BCT, some recruits experience it as highly stressful. A considerable percentage (approximately 20%) of National Guard recruits fail to complete BCT [36]. This suggests that although BCT is a uniform stressor experienced by all recruits, its impact and perceived stressfulness vary among individuals. A study of Swiss Armed Forces recruits participating in a 21-week BCT course found that higher perceived stress at the beginning of the training was associated with greater mental distress and poorer military performance at later stages [37]. Previous studies focusing solely on BCT completers have overlooked valuable data from a significant minority of individuals who exhibit reduced resilience.

To comprehensively understand how BCT influences adaptive functioning and study resilience processes over time, we use a prospective, longitudinal research design with data collection before and after the relatively uniform stressor of BCT. This design makes it possible to capture changes within participants over time and allows us to test the temporal order of variables. Longitudinal designs permit stronger inferences about causal effects, although not with the rigor of a randomized controlled trial. Our design also provides insights into the underlying mechanisms contributing to individual variations in resilience and lays the groundwork for studying the impact of unforeseen events, such as the COVID-19 pandemic and military deployments, on resilience trajectories. By evaluating the perceived frequency and intensity of stressor exposure during BCT, we capture the individual experiences of military recruits. Furthermore, our focus on young, healthy participants at the beginning of their military careers, *before* significant exposure to military stressors, enables us to take a developmental approach to studying resilience within a military context. Finally, the integration of a laboratory substudy within our research design provides an opportunity to conduct in-depth, multilevel assessments of a subset of the cohort. This laboratory substudy allows us to investigate neurobehavioral markers of self-regulation that may predict resilience trajectories, enhancing our understanding of the underlying mechanisms associated with resilience in the context of military training.

### Participants and Recruitment

The enrollment of participants in this study was based on specific inclusion criteria. Individuals aged ≥17 years who were newly enlisted members of the National Guard and had been assigned a ship date to complete BCT during the study period were eligible for participation. Exclusion criteria included a history of prior military service or any previous experience with BCT.

To recruit participants, a consecutive approach was used within designated National Guard Recruitment Sustainment Program (RSP) units across the state until the target sample was reached. The research team recruited participants between April 2019 and October 2021 using briefing presentations held during drill events at military posts. The relevant Army National Guard command provided the research team with a list of all potentially eligible military service members. During the briefings, interested individuals were provided with a study packet, which included a consent or assent letter, a “Help Us Keep in Touch” locator form, a “What to Expect Next” card illustrating the study design in layperson’s terms, and a randomly generated unique study ID number. Throughout the recruitment process, detailed documentation was maintained. This included recording the total number of eligible military service members approached as well as the number of participants enrolled, those who refused to participate, and those found to be ineligible based on the inclusion and exclusion criteria.

### Ethical Considerations

This study was reviewed and approved by the institutional review boards of the UMN (STUDY00004470) and the MVAHCS (VAM-18-00334/1594664). All study procedures were also approved by the relevant military command. All participants provided informed consent. Participants were provided with a letter that explained all the procedures, risks, and benefits. This information was verbally presented during the briefings, and participants indicated their consent to take part in the survey component by entering a unique study ID number to begin the web-based survey. A waiver of the requirement to document consent for the survey component of this study was granted. The survey component of this study was also provided an approval to include individuals aged <18 years under 21 CFR 50.51/45 CFR 46.404. From the subset of participants aged ≥18 years taking part in the laboratory substudy, written informed consent was obtained before participation in any laboratory study procedures. Participants in the longitudinal survey component of the study will be compensated up to US $150 for the completion of all the currently planned survey waves (US $25 for the time-1 survey, US $35 for the time-2 survey, US $40 for the time-3 survey, and US $50 for the time-4 survey). Owing to military regulations, articipants were not compensated for participating in the baseline (time 0) survey. Participants in the laboratory component were compensated up to US $400 for the completion of both the pre-BCT and post-BCT visits.

### Survey Data Collection Procedures

For the web-based survey component conducted at baseline (time 0), participants were asked to complete a battery of self-report questionnaires on Qualtrics as well as select tests from the PCNB. Time-0 data were collected using study Chromebooks (Google LLC) in classrooms at local armories. Participants were instructed to log into a secure web-based Qualtrics platform using their unique study ID for authentication. Most participants completed the baseline data collection procedures within 90 to 120 minutes.

As exposure to BCT is required for continued follow-up, participants are monitored over the course of the study and retrospectively excluded if they are discharged before exposure to BCT. Participants’ exposure to BCT is verified before the initial follow-up wave (time 1), and those eligible to continue study participation are invited to complete the battery of self-report questionnaires at each follow-up time point. All follow-up assessments are completed outside the drill training. Participants complete either a confidential web-based survey of the battery of computerized self-administered questionnaires linked to the participants’ study ID or a paper-and-pencil version of questionnaires marked with the participants’ study ID.

To ensure the continuity of follow-up assessments, we adapted the longitudinal retention model developed by Scott and colleagues [38] to engage and maintain contact with participants from enrollment to the final follow-up time point. During enrollment, participants were asked to provide detailed contact information, BCT ship and return dates, and full contact information for 3 alternate contacts who can help the study team locate the participant if needed. Study engagement will be maintained between follow-up waves through periodic communication (ie, newsletters and greeting cards), and survey nonrespondents will be contacted to encourage survey completion.

### Measures

#### Self-Report Variables

Table 1 lists the self-report questionnaires administered via Qualtrics at baseline and at each follow-up time point. This battery of measures assesses 8 domains (internalizing symptoms, externalizing problems, social-occupational functioning, health, self-regulation processes, supportive relationships, personality, and vulnerability or risk factors). Although most questionnaires are repeated across all time points (time 0 to time 4), as they are critical for tracking outcome trajectories and resilience processes, some are completed only at time 0 (ie, personality) or time 1 (ie, BCT stressor exposure), as they do not require repeated assessment.

**Table 1:** Self-report measures administered over time in the Advancing Research on Mechanisms of Resilience longitudinal study.

Outcome domains and measures			Items, n		Time 0^a^		Time 1^b^		Time 2^c^		Time 3^d^		Time 4^e^
**Internalizing symptoms**													
	PROMIS^f^-Depression 4 Scale [39]	4		✓		✓		✓		✓		✓	
	PROMIS-Anxiety 4 Scale [39]	4		✓		✓		✓		✓		✓	
	PROMIS-Anger 5 Scale [39]	5		✓		✓		✓		✓		✓	
	Primary Care PTSD^g^ Screen for DSM-5^h^ [40]	6		✓		✓		✓		✓		✓	
**Externalizing problems**													
	Behavioral Report on Rule-Breaking Questionnaire [41]	18		✓		✓		✓		✓		✓	
	Alcohol Use Disorders Test [42]	10		✓		✓		✓		✓		✓	
	Drug Abuse Screening Test [43]	10		✓		✓		✓		✓		✓	
**Social-occupational functioning**													
	PROMIS–Role Satisfaction Scale [44]	4		✓		✓		✓		✓		✓	
	PROMIS–Social Participation Scale [44]	4		✓		✓		✓		✓		✓	
	Couple Satisfaction Inventory [45]	4		✓		✓		✓		✓		✓	
	Revised Conflict Tactic Scale [46]	2		✓		✓		✓		✓		✓	
	Health and Work Performance Questionnaire [47]	12		✓		✓		✓		✓		✓	
	Utrecht Work Engagement Scale [48]	9		✓		✓		✓		✓		✓	
**Health**													
	Patient Health Questionnaire-15 [49]	15		✓		✓		✓		✓		✓	
	Graded Chronic Pain Scale-Revised [50]	6				✓		✓		✓		✓	
**Self-regulatory processes**													
	Difficulties in Emotional Regulation Scale-16 [51]	16		✓		✓		✓		✓		✓	
	Multidimensional Experiential Avoidance Questionnaire-30 [52]	30		✓		✓		✓		✓		✓	
	Attention Control Scale [53]	20		✓		✓		✓		✓		✓	
	Flexible Regulatory Emotional Expression Scale [54]	16		✓		✓		✓		✓		✓	
	Identity Coherence Scale [55]	12		✓		✓		✓		✓		✓	
	Response to Stressful Experiences Scale [56]	22		✓		✓		✓		✓		✓	
**Supportive relationships**													
	PROMIS-Emotional Support 4 Scale [57,58]	4		✓		✓		✓		✓		✓	
	PROMIS-Informational Support 4 Scale [57,58]	4		✓		✓		✓		✓		✓	
	PROMIS-Instrumental Support 4 Scale [57,58]	4		✓		✓		✓		✓		✓	
	DRRI-2^i^ Unit Support Scale [59]	12		✓		✓		✓		✓		✓	
**Personality**													
	Multidimensional Personality Questionnaire-Brief Form [60]	155		✓									
	Triarchic Psychopathy Measure [61]	58		✓									
**Vulnerability or risk factors**													
	Adverse Childhood Experiences Questionnaire [62]^j^	17		✓						✓			
	DRRI-2 Prior Stressors Scale [59]	18		✓									
	DRRI-2 Adapted Postdeployment Life Stressor Scale [59]	14				✓		✓		✓		✓	
	Suicidal Ideas and Behaviors Questionnaire [63]	6		✓									
	Basic Training Stressors Scale [23]	14				✓							

^a^Baseline, before basic combat training.

^b^2 weeks after basic combat training.

^c^6 months after basic combat training.

^d^12 months after basic combat training.

^e^18 months after basic combat training.

^f^PROMIS: Patient Reported Outcomes Measurement Information System.

^g^PTSD: posttraumatic stress disorder.

^h^DSM-5: Diagnostic and Statistical Manual of Mental Disorders, Fifth Edition.

^i^DRRI-2: Deployment Risk and Resilience Inventory-2.

^j^Adverse Childhood Experiences Questionnaire adapted for youths at time 0.

#### Outcomes

Primary outcomes include trajectories of internalizing symptoms, externalizing problems, social-occupational functioning, and global adaptive functioning. Secondary outcomes are self-reported self-regulatory processes, neurobehavioral markers of self-regulation, and ecologically valid markers of functioning extracted from administrative military records.

#### Neurocognitive Measures

Table 2 lists the neurobehavioral functions and major cognitive domains assessed by tests from the PCNB administered at baseline. Neurocognitive tests measure performance accuracy (eg, proportion of correct responses) and speed (eg, median correct response time) in major cognitive domains [64].

**Table 2:** Neurobehavioral functions and domains assessed using tests from the Penn Computerized Neurocognitive Battery at baseline in the Advancing Research on Mechanisms of Resilience longitudinal study.

Neurobehavioral Function	Domain	Test
Executive control	Attention	Penn Continuous Performance Test
Complex cognition	Language reasoning	Penn Verbal Reasoning Test
Episodic memory	Face memory	Penn Facial Memory Test
Social cognition	Emotion differentiation	Penn Emotion Differentiation Test

### Laboratory Substudy Procedures

A subsample of the longitudinal cohort (123/1201, 10.24%) was recruited for a laboratory visit, both before and after BCT. Laboratory participants were selected based on their responses to baseline self-report measures and a predictive algorithm developed in the pilot UG3 phase of this project (details reported elsewhere; S Noorbaloochi et al, unpublished data, 2023). This strategy was intended to provide a subsample enriched with participants at a relatively high risk of maladaptive functioning (~72/123, 58.5%) compared with a low-risk group (~48/123, 39%). Participants were briefly screened via phone call, and those reporting contraindications to MRI due to the potential presence of metal in the body (eg, participants employed as a metal worker or participants with implanted medical devices) or due to problems with being in enclosed places were excluded.

Data for the laboratory substudy were collected during an 8- to 9-hour combined pre-BCT laboratory visit to the MVAHCS and UMN Center for Magnetic Resonance Research and during a 4- to 5-hour post-BCT laboratory visit at the MVAHCS. During the pre-BCT and post-BCT laboratory visits, participants completed a structured clinical interview (1-2 hours) with a trained master’s level assessor under the supervision of a licensed clinical psychologist (CRE or PAA). The Structured Clinical Interview for Diagnostic and Statistical Manual of Mental Disorders, Fifth Edition (DSM-5) was used to diagnose lifetime mental disorders and determine whether the disorder is “current” [65]. The Clinician-Administered PTSD Scale for DSM-5 was used to diagnose PTSD [66]. Participants also completed a battery of self-report measures assessing constructs related to self-regulation (Table 3).

**Table 3:** Self-report measures administered in the Advancing Research on Mechanisms of Resilience laboratory substudy before and after basic combat training (BCT).

Measure	Items, n	Pre-BCT laboratory visit	Post-BCT laboratory visit
Patient Health Questionnaire-9 [67]	9	✓	✓
Beck Depression Inventory-II [68]	21	✓	✓
Depressive Symptom Index-Suicidality Subscale [69]	4	✓	✓
State Trait Anxiety Inventory [70]	40	✓	✓
Positive and Negative Affect Schedule [71]	20	✓	✓
Multidimensional Experiential Avoidance Questionnaire [72]	62	✓	✓
Anxiety Sensitivity Index [73]	16	✓	✓
Intolerance of Uncertainty Scale-Short Form [74]	12	✓	✓

Participants were asked to complete an EEG session to measure resting-state neural function as well as brain responses during cognitive tasks. We used electrodes embedded in an elastic cap to record electrical activity from 128 scalp sites. The precise physical location of the electrodes was recorded in 3D space with respect to auricular and nasion landmarks so that EEG recordings could be integrated with the corresponding structural MRI data. To measure eye movements for the detection of bioelectrical artifacts, vertical electro-oculograms were recorded from above and below the right eye, and horizontal electro-oculograms were recorded from near the outer ocular canthi. Left and right forearm electromyographs and electrocardiograms were recorded to identify and reduce artifacts and quantify the aspects of muscle contraction associated with button presses. EEG signals were digitized at the rate of 512 Hz with 0.5-200 Hz low frequency and 200 Hz high frequency bandpass filters. Each participant completed assessments of visual acuity and handedness as well as provided information about medications, alcohol consumption, caffeine consumption, and sleep in the last 24 hours. Self-report ratings of emotional state were assessed before and after EEG recording sessions using the Positive and Negative Affect Scale [71].

At the pre-BCT laboratory visit only, MRI data were collected at the UMN’s Center for Magnetic Resonance Research on a 3T (Siemens) Prisma scanner using a 32-channel birdcage head coil with foam pads to minimize head movements. Sequences included T1-weighted Magnetization Prepared-Rapid Gradient Echo, T2-weighted Sampling Perfection with Application optimized Contrast using different flip angle Evolution, diffusion-weighted MRI, resting-state functional MRI (fMRI), and task-based fMRI.

### Neurobehavioral Assessments

#### Overview

During the EEG session at the pre- and post-BCT visits, participants were asked to perform a series of neurobehavioral tasks applying paradigms to assess attentional control (dot-probe paradigm) [75,76], emotional inhibitory control (go or no-go paradigm) [77,78], and feedback processing (gambling decision paradigm) [79]. In addition, participants were asked to complete a video game–based task applying the Pavlovian-instrumental generalization paradigm [80] to assess adaptive versus maladaptive avoidance in the face of a physical threat.

#### Dot-Probe Task

Attentional bias is assessed using the dot-probe task [75,76]. Participants passively inspect rapidly presented face pairs with neutral, happy, or angry and threatening expressions. The face pairs disappear and are replaced by a blank space on one side of the screen and an asterisk (target probe) on the other. Participants are instructed to respond as quickly as possible to indicate the side on which the target probe appeared.

#### Emotional Go or No-Go Task

Inhibitory control is assessed using the emotional go or no-go task [77,78]. Participants view a series of rapidly presented visual targets and nontargets. Participants are instructed to press a response button for each target presented (“go trials”) and to avoid pressing the button for each nontarget (“no-go trials”). Errors (response to no-go trials or failure to respond to go trials) are accompanied by visual (red bar in the center of the screen) feedback. The task is completed in 3 blocks with adaptive response timing windows to increase task difficulty based on performance. Participants are provided with visual feedback on task points earned or lost, which is displayed on the screen every 20 trials. During the second block, time windows are shortened to increase the task’s difficulty just beyond what is feasible for participants. Points awarded during the second block for correct responses are also significantly reduced.

#### Gambling Decision Task

Error and performance monitoring is measured using the gambling decision task [79]. For each trial, participants are presented with 2 adjacent squares, each enclosing a number (5 or 25). Participants are instructed to choose between the 2 squares and then receive feedback (the chosen box turns red or green to signify either a win or a loss, with the winning and losing colors counterbalanced across participants). Winning and losing squares are preprogrammed in a randomized manner such that participants must make guesses and will be unable to detect a true pattern using the task feedback. Thus, the task assesses participant biases toward risk-taking following the experience of loss.

#### Farmer Task

While in the MRI environment, participants completed the farmer task [80]. In this gamified task, the participant is asked to take on the role of a farmer whose task is to travel between a shed and garden to harvest crops. Two different roads connect the shed to the garden: (1) a short road and (2) a long road. Traveling the short road is perilous (contingently associated with an electric shock; 3-5 mA for 100 ms) but allows the farmer quick travel from shed to garden, thereby assuring a successful harvest. Conversely, traveling the long road is always safe (never associated with shock) but often prevents the farmer from arriving at the garden before “wild birds” eat the crop.

Before each trial, a stimulus is presented to the participant that they learn can indicate whether the short road will deliver the electric shock. Specifically, participants learn, through fear conditioning, that shock is predicted by a visual ring of extreme size (conditioned danger cue: CS+) but not by rings of the smallest size (conditioned safety cue: CS–) displayed on the screen. During the game, rings of intermediary sizes, presented in the absence of a shock, serve as generalization stimuli. The task captures fear generalization and subsequent instrumental avoidance by assessing task-based behaviors in response to generalization stimuli that are similar (but not identical) to CS+. Decisions to “unnecessarily” avoid during safe cues is considered *maladaptive* because danger (threat of shock) is not a realistic possibility, and avoiding unnecessarily compromises performance on the task. Adaptive responding is operationalized by higher rates of decisions to push through (ie, low levels of avoidance) when encountering safe yet danger-resembling cues.

### DNA Samples

All participants enrolled in the longitudinal study were asked to provide a DNA saliva sample at baseline, which is stored for future analysis. Participants in the laboratory substudy are also asked to provide a blood sample to be stored for future research.

### Administrative Data

The study team will work with the local National Guard to extract administrative data capturing sociodemographic and service-related variables (ie, Armed Forces Qualification Test score, training dates, and military discharge). Administrative data from military records will be deidentified and matched by participant ID.

### Sample Size and Power Analysis

Sample size was calculated based on the primary objective of the study (aims 1 and 2) using structural equation models of various hypothesized structures (nested and longitudinal). This demand resulted in a sample size that could provide sufficient power to test such hypotheses across varying df. Control of root mean square error of approximation (RMSEA) was the criteria used for our sample size and power calculations. According to MacCallum and colleagues [81], RMSEAs of 0.01, 0.05, and 0.08 indicate excellent, good, and mediocre fit, respectively. Table 4 presents the required sample sizes and the corresponding powers for df=2 and df≥5, assuming a null RMSEA of 0.05 and an α of .05. Assuming a retention rate of 65% to reach an effective sample size of 780, we needed to recruit 1200 participants. Note that for n=780, structural equation models with df≥5 would have an expected power of at least 0.909. The column for df=2 demonstrates that for df smaller than 5, the power for our feasible sample size drops below conventional 0.80.

**Table 4:** Power estimates (chi-squared test for root mean square error of approximation=0.05).

Sample size	Estimate (*df*)	Estimate (*df*)
700	0.567 (2)	0.878 (5)
800	0.619 (2)	0.916 (5)
1000	0.707 (2)	0.961 (5)
1200	0.776 (2)	0.982 (5)

### Data Management

Data collected via the web-based survey platform (Qualtrics) administered at the UMN will be downloaded securely into the study databases stored on a shared server at the MVAHCS, with access limited to authorized study personnel. Qualtrics includes a complete suite of features to support Health Insurance Portability and Accountability Act compliance, including a full audit trial, user-based privileges, and integration with the institutional Lightweight Directory Access Protocol (LDAP) server. Hard copy surveys will be double entered and checked for consistency. Laboratory substudy data (ie, neuroimaging and EEG data) will be stored in secure databases held on servers at the UMN and will be available only to lead investigators of each substudy component and the principal investigator. Administrative data will be transferred directly from the Minnesota Army National Guard to a secure server at the MVAHCS via secure data transfer. At the MVAHCS, the data will be accessible by a named Center for Care Delivery and Outcomes Research (CCDOR) Data and Statistics Team member. This individual will deidentify the personal information, match records with the study ID number, and transfer them to a shared server for the study. The study ID numbers will be used for data transfer, communication, and analysis purposes to protect confidentiality. Participant contact information linked with the study ID number will be stored in an SQL server database that will be created on internal Department of Veteran Affairs (VA) web servers. Only individuals with a need to access the data, as vetted by the principal investigator, will be granted access. Access to contact information will be obtained through Windows authentication (ie, personal identity verification card and password for the network).

### Statistical Methods

Aim 1 will characterize adaptation among young military recruits over the first 2 years of service. Consistent with a hierarchical concept of the structure of resilience, we will use latent growth mixture modeling to identify the latent trajectories of adaptation and its indicators. We will apply latent growth mixture modeling at the level of internalizing symptoms, externalizing problems, and social-occupational functioning as well as an overall model that simultaneously embeds all 3 domains into a model of adaptive functioning. We hypothesize that at least three trajectories (one showing resilient adaptive functioning or low pathology and the other two showing new-onset and chronic distress) will emerge for each domain assessed (internalizing symptoms, externalizing problems, social-occupational functioning, and global adaptive functioning).

Note that all remaining analyses for aims 2 and 3 will be conducted on the trajectory class membership data for each of these 4 domain outcomes to test for the specificity of resilience mechanisms***.***

Aim 2 will identify the promotive factors and protective processes contributing to individual variations in adaptation. We will apply structural equation modeling and Bayesian network analysis to detect and identify the latent constructs and the dependency structure between different domains. We will also examine the effects of protective and vulnerability factors on membership in different trajectory classes. We propose to investigate this in 2 complementary ways. First, in our structural equation modeling, we will augment the measurement submodels of the most parsimonious latent growth structural mixture models accepted in aim 1 with the indicators of protective and vulnerability measurements (Tables 1 and 2) and evaluate the change in the relationships (coefficients) that indicators have with the latent trajectories. We will treat potential predictors as moderators or mediators, depending on their hypothesized role in the model, and we will test their contribution to the model by change in model fit indices using the Akaike information criteria (AIC), the Bayesian information criteria (BIC), root mean squared error (RMSE), and goodness-of-fit (G^2^) also known as the likelihood ratio test. Second, the trajectory class membership assignment in aim 1 will be used as dependent variables (resilient vs nonresilient or distressed trajectories), and protective and vulnerability factors will be used as predictors in random intercept multinomial logistic mixed models that will provide a quantitative picture of the unique contribution of these measures. We hypothesize that the risk factors listed in Tables 1 and 2 will predict membership in nonresilient or distressed trajectory classes, whereas protective or promotive factors will predict membership in the resilient or nondistressed trajectory classes. All analyses will be estimated within the propensity classes that adjust for any possible lack of imbalance and will be performed on both complete cases and 5 imputed data sets.

Aim 3 will investigate whether neurobehavioral markers of self-regulation assessed using a series of performance-based tasks involving EEG and fMRI assessments are predictive of resilient and nonresilient trajectories. EEG recordings will be put through an independent component analysis–based processing pipeline to isolate and remove noisy time segments, signal artifacts (eg, eye movements), and bad electrodes. Recording epochs centered around the onset of task stimuli will be extracted for the analysis of event-related potentials and time-frequency energy. Given that this study focuses on conflict monitoring and bottom-up covert attention, we plan to examine brain responses at midline frontal (eg, FCz) and lateralized posterior electrodes (eg, PO7 and PO8), respectively. We will quantify brain response metrics such as feedback-related negativity and θ-band (4-8 Hz) oscillations, which we predict will be associated with trajectory class membership. fMRI data collected during the farmer task will be analyzed using an event-related design to model blood oxygenation level dependent (BOLD) fluctuations due to stimuli onsets using Analysis of Functional Neural Images software [82]. Functional activation maps will be computed by regressing each voxel’s fMRI response time course onto an ideal response function for 5 stimulus types: the danger cue, 3 classes of safety cues with parametrically varying levels of perceptual similarity to the danger cue, and a control condition including a safety cue with no perceptual similarity to the danger cue [83]. We will focus particularly on fMRI responses to these stimuli in brain areas associated with fear reactivity (anterior insula and dorsomedial prefrontal cortex) and fear inhibition (ventromedial prefrontal cortex and anterior hippocampus). Fear-related brain activations to safety cues resembling danger cues (relative to safety cues without danger-cue resemblance) will be used as predictors of maladaptive avoidance (costly or unnecessary avoidance of safe cues perceptually resembling danger cues). Finally, these brain and behavioral responses, as well as their moderation by protective (eg, social support, military cohesion, and self-regulation) and vulnerability factors (eg, BCT stressors and anxiety traits), will be used as predictors of trajectory class membership.

### Handling of Missing Data

Every effort will be made to maximize the completion of data and responding across waves in the longitudinal survey (aims 1 and 2). However, the data collected at each wave will be subject to various random missingness. At the end of each wave, the mechanism of missingness for each measured variable will be assessed, and an imputation model appropriate to that variable (predictor, response, or adjustor covariate) will be identified. Appropriate imputation methods based on variable characteristics (eg, linear regression for continuous variables, multinomial logit models for categorical models, and propensity matching based on multiple predictors) will be used. After selecting a relevant model and, more importantly, deciding on the predictors of missingness, 5 imputed data sets will be constructed for each wave. The relevant analyses will then be based on the combined inferences derived from these 5 imputed data sets.

## Results

This UG3/UH3 project was reviewed by the National Institutes of Health (Multimedia Appendix 1) and initially funded in August 2017. The UG3 pilot phase for establishing the feasibility of the larger prospective longitudinal study was conducted between August 2017 and December 2018 and is reported elsewhere [23]. The UH3 phase of the project was funded in March 2019. Study enrollment began on April 14, 2019, and ended on October 16, 2021. A total of 1201 participants are enrolled in the study; follow-up data collection is ongoing and projected to continue through February 2024.

## Discussion

### Aims of the Study

ARMOR targets a gap in resilience research by providing a rich longitudinal database to assess resilience as a dynamic process of successful adaptation in response to significant adversity and identify the processes underlying resilience. Specifically, this study will characterize the trajectories of adaptation (ie, resilient vs nonresilient) among young US Army National Guard recruits in response to BCT beginning at career onset and spanning 2 subsequent years (aim 1) and identify promotive factors and protective processes contributing to individual variations in adaptation (aim 2). ARMOR will also investigate whether neurobehavioral markers of self-regulation are predictive of resilient versus nonresilient trajectories (aim 3).

### Strengths and Limitations

This study takes a longitudinal, multilevel approach to examine the mechanisms underlying individual variations in resilience trajectories. Through the assessment of recruits before BCT and follow-up across 4 time points after BCT, the design provides an opportunity to map individual trajectories in response to exposure to a naturally occurring, uniform military challenge. Although previous research has identified numerous resilience factors, most existing longitudinal studies have relied almost entirely on self-report. This study uses an embedded laboratory substudy design that integrates novel laboratory-based experimental paradigms for assessing dynamic self-regulatory processes into a large, prospective, and longitudinal study. This approach will enable us to explore whether neurobehavioral markers of self-regulation assessed using a series of performance-based tasks involving EEG and fMRI assessments are predictive of resilient versus nonresilient trajectories. A final strength is the involvement of military stakeholders in the study design. Beginning at the early planning stage, members of the local National Guard command were involved in the research process, providing feedback on relevant research questions and consultation on the feasibility of the study methods. The selection of the outcome measures was informed by their priorities and military experience. The local National Guard command also facilitated the study team’s access to military personnel and allocated training time for the investigators to present information about the study to potential participants and collect baseline data.

Despite these strengths, ARMOR has several limitations. The nature of our study design, incorporating in-person laboratory visits for a subset of the cohort, required that participants reside within a limited geographical area. Therefore, it was necessary to limit the recruitment of participants to the National Guard of a single state. Although we will investigate the influence of demographic characteristics on our findings, the results may not be generalizable to all military service members from other states or to all military branches.

Although we attempt to be exhaustive in our assessment of the risk and protective processes relevant to resilience in the context of military stress, it is possible that some relevant variables may not be assessed. For example, we recognize that collecting data from other informants, such as peers, commanders, or family members, would provide information on the influence of environmental factors on resilience. However, collecting such data would be prohibitive in terms of cost and time given the number of participants we are studying. However, we obtained permission from military service members to access military and VA administrative records so that these sources of information (eg, ecologically valid markers of functioning from administrative military records) can be accessed and analyzed in the future.

A key challenge for any longitudinal study is the retention of participants over time. This can be particularly difficult when participants are both young and highly mobile, as is the case in this study. We have taken steps to ensure a satisfactory response rate so that the study will be adequately powered to detect small effects in analyses testing our major hypotheses. Although follow-up survey data collection continued throughout the COVID-19 pandemic, all in-person research activities, including participant recruitment, baseline data collection, and laboratory visits, were suspended from March 2020 to early August 2020. During this period, some participants returned from BCT but were unable to complete in-person laboratory visits.

As of March 2020, over half (755/1201, 62.86%) of the ARMOR participants had been recruited and assessed at baseline before the pandemic outbreak. All remaining participants were enrolled and completed baseline measures after the pandemic outbreak between August 2020 and October 2021. This presents a rare opportunity to conduct a “natural experiment” in which recruits who have completed the stressful but potentially resilience-enhancing BCT before the pandemic can be compared with those who have not. For details outlining the collection of additional COVID-19–focused surveys through supplemental funding from the National Center for Complementary and Integrative Health, see the Multimedia Appendix 2 [1,31,38,44,45,48,84-127].

### Conclusions

The ARMOR study provides a rich data set to identify the predictors and mechanisms of resilient and nonresilient outcomes in the context of military stressors. Understanding the neurobiological, cognitive, and social mechanisms underlying adaptive functioning following military stressor exposure is essential for enhancing the resilience of military service members. The findings from our study will facilitate the understanding of the mechanisms underlying self-regulatory processes implicated in resilience and thus provide a foundation for the development of prevention and intervention strategies that help to promote positive adaptation and resilience among young military recruits. Specifically, the findings of this study may support the optimization of mindfulness-based interventions, which focus on increasing the awareness of one’s thoughts, emotions, and actions to improve specific aspects of executive functioning, including attention, cognitive control, and emotion regulation.

## Appendix

Multimedia Appendix 1 Summary statement from National Institutes of Health review.

Multimedia Appendix 2 Advancing Research on Mechanisms of Resilience (ARMOR) study COVID-19 supplemental protocol.

## Abbreviations

AIC: Akaike information criteria
ARMOR: Advancing Research on Mechanisms of Resilience
BCT: basic combat training
BIC: Bayesian information criteria
BOLD: blood oxygenation level dependent
CCDOR: Center for Care Delivery and Outcomes Research
DSM-5: Diagnostic and Statistical Manual of Mental Disorders, Fifth Edition
EEG: electroencephalography
fMRI: functional magnetic resonance imaging
G^2^: goodness-of-fit
LDAP: Lightweight Directory Access Protocol
MRI: magnetic resonance imaging
MVAHCS: Minneapolis Veterans Affairs Health Care System
PCNB: Penn Computerized Neurocognitive Battery
PTSD: posttraumatic stress disorder
RMSE: root mean squared error
RMSEA: root mean square error of approximation
RSP: Recruitment Sustainment Program
UMN: University of Minnesota-Twin Cities
VA: Veteran Affairs

## Data Availability

Data sharing is not applicable to this paper, as it does not report on any data generated or analyzed during this study.
