# Supplementary material for: Adaptation in Young Military Recruits: Protocol for the Advancing Research on Mechanisms of Resilience (ARMOR) Prospective Longitudinal Study
Source: JMIR Res Protoc. 2023 Oct 4;12:e51235. doi: 10.2196/51235 (PMC10585449; doi:10.2196/51235)
Supplement: Multimedia Appendix 1 [file resprot_v12i1e51235_app1.pdf]

SUMMARY STATEMENT

PROGRAM CONTACT: (Privileged Communication) *Release Date:* 03/23/2017

Dr. Lanay Mudd
301-594-9346
muddlm@mail.nih.gov

Revised Date:

Application Number: 1 UG3 AT009651-01
Formerly: 1UG3MH114301-01

Principal Investigators (Listed Alphabetically):

ERBES, CHRISTOPHER R
POLUSNY, MELISSA A. (Contact)

Applicant Organization: UNIVERSITY OF MINNESOTA

Review Group: ZRG1 RPHB-R (51)
Center for Scientific Review Special Emphasis Panel
PAR 16-326: Advancing Basic Behavioral and Social Research on Resilience

Meeting Date: 03/03/2017
Council: MAY 2017
Requested Start: 07/01/2017
RFA/PA: PAR16-326
PCC: MUDDL

Project Title: Advancing Research on Mechanisms of Resilience (ARMOR): Prospective Longitudinal Study of Adaptation in New Military Recruits
SRG Action: Impact Score:34
Next Steps: Visit https://grants.nih.gov/grants/next_steps.htm
Human Subjects: 30-Human subjects involved - Certified, no SRG concerns
Animal Subjects: 10-No live vertebrate animals involved for competing appl.
Gender: 1A-Both genders, scientifically acceptable
Minority: 1A-Minorities and non-minorities, scientifically acceptable
Children: 3A-No children included, scientifically acceptable
Clinical Research - not NIH-defined Phase III Trial

Project Year	Direct Costs Requested	Estimated Total Cost
1	499,959	629,948
2	499,771	629,711
3	499,833	629,790
4	499,820	629,773
5	499,937	629,921
TOTAL	2,499,320	3,149,143

1UG3AT009651-01 POLUSNY, MELISSA

RESUME AND SUMMARY OF DISCUSSION: This significant application will conduct a longitudinal study of National Guard recruits before, at conclusion, and later after Basic Combat Training (BCT). If successful, the longitudinal research could identify trajectories of adjustment and predictors of resilience to stress. A sub-sample examined using neuroimaging and EEG pre-BCT/post-BCT may identify neurological pathways of self-regulation underlying resilience. This is a strong team who are a good match for the project, with requisite expertise and a history of collaboration. During the discussion, the panel noted several strengths of the application, including the solid baseline and multiple longitudinal measurements related to meaningful milestones for UG3 success. The research design is very rigorous and innovative, as is the focus on self-regulation. However, the neurobiological support developed in the scientific premise is weak and the integration with EEG and neuroimaging lacks consistency throughout. Some concern was expressed about the absence of a direct measure of coping and, further, that consideration of individual differences in personal adoption of the National Guard's "meaningfulness of mission" is not included in the model. Apparently, sex/gender differences will not be addressed by modeling. Nor is generalizability of results to other groups given adequate attention in the application. There were some different views expressed by the panel on the high or moderate level of innovation. There are no human subject concerns but, while not required, a plan is not included for handling incidental findings and disclosures made during imaging and assessment procedures may be appropriate. Nevertheless, overall, the strengths outweigh the weaknesses of the application.

DESCRIPTION (provided by applicant): Resilience is defined as the capacity to adapt successfully through multiple processes in response to adverse events. While explanatory models are still in their infancy, theoretical and empirical evidence suggest that self-regulatory processes may underlie resilient functioning. This proposal will develop (Phase 1, Year 1) and then implement (Phase 2, Years 2-5) a study of 1,200 National Guard recruits, in the first two years of their enlistment, in order to identify patterns and processes of adaptive (resilient) functioning in the face of Basic Combat Training (BCT) and other adverse events. Aims: The aims for Phase 1 are focused on planning and preparing for a longitudinal study of resilience in National Guard recruits and include: 1) Use prior datasets to develop statistical algorithms for selecting pilot baseline data collection procedures to determine feasibility of enrollment and response rate targets for longitudinal study, 3) Develop and refine contextualized measures of National Guard training stressor exposure, and 4) Evaluate the feasibility and acceptability of neurobehavioral markers of resilience processes. During Phase 2, a comprehensive model of resilience will be investigated using a four-year, mixed-method, longitudinal study to participants with high and low probabilities of resilience: 1) Characterize latent trajectories of adjustment among new military recruits beginning prior to BCT over two years, 2) Identify promotive and protective predictors of resilient functioning in National Guard soldiers, and 3) Investigate neurobehavioral pathways of self-regulation predictive of resilience trajectories in new military recruits. Procedures and Analyses: In Phase 1, prediction algorithms will be established using analysis of datasets gathered in prior National Guard studies. The measurement of BCT stressors, and feasibility of Phase 2 recruitment plans, will involve online surveys of 100 National Guard recruits before, during and immediately after BCT. The feasibility and acceptability of neurobiological measures through imaging and laboratory tasks piloting procedures will be assessed with 10 recruits undergoing procedures before and after BCT. In Phase 2, on-line self-reports of symptoms (depression, anxiety, alcohol or drug use), adaptive social functioning, and promotive, protective, and risk factors will be collected before and at 4 time points after BCT with 1200 National Guard recruits. Growth mixture modeling will establish resilient and non-resilient trajectories of adaptive functioning. Aim 2 will use predictive modeling of trajectories using predictors collected during the baseline and subsequent time points. In Aim 3, 120 recruits will complete laboratory tasks (Farmer's task, go/no-go task, dot probe task, performance monitoring task) before and after BCT while undergoing MRI or EEG scans. Analyses will determine if these tasks can

discriminate between recruits in the resilient vs. non-resilient trajectories of functioning established in Aim 1.

PUBLIC HEALTH RELEVANCE: Resilience is the capacity for positive adaptation in the context of significant stress or adversity. This project will enhance our understanding of how self-regulation leads to resilience by studying National Guard soldiers over the first two years of service. Findings from the study can help develop new ways to enhance resilience in people like military service members or first responders who are at high risk for exposure to adverse situations.

CRITIQUE 1

Significance: 2
Investigator(s): 1
Innovation: 4
Approach: 6
Environment: 2

Overall Impact: This is a UG3/UH3 application that proposes to assess military recruits longitudinally, before and after basic combat training, to assess resilience (i.e., capacity for positive adaptation under stress/adversity). This is a significant proposal that will assess recruits prior to a challenge (basic combat training) and follow them longitudinally to determine trajectories of resilient vs non-resilient adaptation. The proposal contains notable strengths including a strong research team, well-developed Phase 1 approach, innovative longitudinal approach, and an impressive environment to support the proposed project. However, weaknesses include inconsistencies and ambiguities in the Phase 2 methods for neuroimaging and psychophysiology, as well as the relatively limited integration of these aspects of the project into the larger aims of the proposal and conceptualization of resilience.

1. Significance:

Strengths

- Proposal addresses an important issue, resilience in military recruits, which may offer important insights into resilience in other populations as well.
- Strong justification for the scientific premise was provided in the form of extant literature and the research team’s prior work.
- Well-developed conceptual model of resilience based on team’s prior work.

Weaknesses

- Limited integration of concepts of resilience, regulation, and underlying neurobiological processes of interest.

2. Investigator(s):

Strengths

- A strong research team with expertise in resilience, social function, cognitive neuroscience, neuroimaging, statistics, and longitudinal research has been brought together.
- Multiple Principal Investigator team has strong record of successful prior funding and publication of prior work.

Weaknesses

- None noted.

3. Innovation:

Strengths

- Innovative approach that assesses military recruits longitudinally, before and after a real-life challenge, basic combat training.

Weaknesses

- MRI data will only be collected pre-BCT and there was limited description of a plan to integrate multi-modal data (e.g., EEG, DTI, and resting-state and task-based fMRI) more thoroughly into the larger approach to this study.

4. Approach:

Strengths

- Relatively rigorous use of Phase 1 to assemble and train the team, design and pilot test procedures, finalize measures/procedures, and transition to Phase 2.
- Several meaningful and well-described milestones were included to justify transition to Phase 2.
- Consideration of missing data and approach for addressing missing data was described.

Weaknesses

- Thresholds for successfully meeting milestones for transition from Phase 1 to Phase 2 were not clearly established.
- It is not clear whether/how BCT is staggered to permit the laboratory assessment (including neuroimaging and psychophysiology) of 120 participants in a 2-week period prior to BCT. Without a staggered approach the lab assessment of 120 participants in 2 weeks does not seem feasible.
- Scientific rigor and transparency of the laboratory assessment and analyses in Phase 2 is weakened by methodological inconsistencies across sections of the proposal, and ambiguities in the samples completing MRI, EEG, pre vs. post assessments, and omission of important details. These issues raise concerns about reproducibility.
- MRI assessments only completed prior to BCT. Limited description of second level analyses of resting state and diffusion weighted neuroimaging analyses. No clear plan to integrate multi-modal imaging data is presented.

5. Environment:

Strengths

- Excellent facilities at the Minneapolis VA RINGS lab for recruitment, interviews, questionnaires, and cognitive assessments.
- The Davenport lab contains the necessary equipment to complete the proposed EEG project.
- Project will use a state-of-the-art 3T Prisma from Siemens.

Weaknesses

- Limited description of the neuroimaging facilities, availability of ancillary devices, and facility access.

Protections for Human Subjects:

Unacceptable Risks and/or Inadequate Protections

- A plan for managing incidental findings was not discussed.

Data and Safety Monitoring Plan (Applicable for Clinical Trials Only):

Not Applicable (No Clinical Trials)

Inclusion of Women, Minorities and Children:

- Sex/Gender: Distribution justified scientifically
- Race/Ethnicity: Distribution justified scientifically
- For NIH-Defined Phase III trials, Plans for valid design and analysis: Not applicable
- Inclusion/Exclusion of Children under 18: Excluding ages <18; justified scientifically
- Children will not participate in this study, and the distribution of race/ethnicity and sex/gender were discussed. All were scientifically justified.

Vertebrate Animals:

Not Applicable (No Vertebrate Animals)

Biohazards:

Not Applicable (No Biohazards)

Budget and Period of Support:

Recommended budget modifications or possible overlap identified:

- Budget requests support for 120 (presumably 60 pre- and 60 post-BCT) MRI scans (and maybe participant compensation), while research approach states post-BCT MRI will not be completed.

CRITIQUE 2

Significance: 3
Investigator(s): 1
Innovation: 3
Approach: 5
Environment: 2

Overall Impact: This is a well-formulated proposal examining self-regulatory processes as a central mechanism contributing to resilience in response to a stressor experience. The stressor utilized is basic training in the Minnesota National Guard. The components of resilience in terms of cognitive factors that contribute to self-regulation are well described, and will be examined through survey, neuropsychological testing, neuroimaging, and EEG. Personality traits such as positive and negative affectivity, and social support will also be examined as predictors of resilience. UG3 will field test baseline data collection procedures, and be involved with planning and pilot studies. UH3 will involve examination of a prospective longitudinal cohort study of new military enlistees assessed prior to and over a period of 24 months following basic training. The investigators are well-experienced with the proposed methodologies and with the proposed participants. The application pays particular attention to scientific rigor and reproducibility. The statistical approaches are well described and appropriate to the

application. The environment is very supportive. This application could potentially have a strong impact in an understanding of self-regulatory processes that are involved in resilience. As this is largely a healthy late-adolescent population, generalizability to older adults or adults suffering from chronic medical conditions is not known.

1. Significance:

Strengths

- Mental health problems, and conduct problems are a longstanding and costly problem for the military. Military recruits are a high-risk group faced with intense stressors. Thus, there is a strong scientific premise for this study. This application combines multiple approaches to measuring resilience (survey, neuropsychological testing, neuroimaging, and EEG). Although other investigators have examined resilience from a variety of these perspectives, the present application is innovative in its combination of the various elements into a unified model of resilience. This project, if successful, will develop and evaluate a unified model of resilience that can then serve as the basis for developing new and potentially more effective programs for enhancing resilience. Thus, the application has strong potential public health significance.

Weaknesses

- A sense of meaning and/or spirituality is a source of resilience that is frequently investigated. Meaning/spirituality does not figure into the current model, but may be an important factor for participants. As such, the model may not be as comprehensive as it could be.

2. Investigator(s):

Strengths

- This is a very strong group of investigators who have expertise in imaging and EEG procedures. The Principal Investigator has expertise in trauma and has had substantial experience with large-scale longitudinal studies with military personnel. She collaborates with a team of experts in imaging and EEG, particularly as related to anxiety and trauma.
- Track record of expertise of investigators is a strength.

Weaknesses

- None noted.

3. Innovation:

Strengths

- The use of novel laboratory based experimental paradigms for assessing self-regulatory processes within the context of a large, prospective, longitudinal cohort study.
- The authors state that the examination of neural mechanisms underlying self-regulatory processes involved in resilience that could be targeted by mind-body interventions such as mindfulness has not previously been conducted.
- National Guard is understudied, and yet provides an excellent opportunity for the study of resilience.
- Development of a unified approach to resilience including self-regulation components, as well as affective and social components is novel.

Weaknesses

- Other labs have examined self-regulatory processes involved in resilience.

4. Approach:

Strengths

- Access to a large population of individuals undergoing a uniform stressor for a discrete period of time is a great strength.
- Previous success in recruiting and working with this population.
- Combination of multilevel methods - survey, imaging, EEG, and neuropsychological testing.
- Scientific rigor and reproducibility are enhanced with strong justifications for statistical power and sample size.
- Transparency is enhanced by methods that are clearly described.
- Scientific premise builds on decades of high quality research from multiple laboratory studies.
- Excellent description of statistical modeling.

Weaknesses

- A sense of meaning and/or spirituality is a frequently named source of resilience. Meaning/spirituality does not figure into the current model, but may be an important factor for participants. As such, the model may not be as comprehensive as it could be.
- Men and women often have very different experiences in the military, and differences are reported in levels of abuse, and attitudes towards men and women. There is no attention to potential differences in the dependent or independent variables by sex and the issue of whether analyses will adjust for sex or whether sub-analyses be conducted is not addressed. It is not clear whether there would be adequate power to address this issue. By lumping both men and women together in analyses, blurring of important responses to the challenge might occur.
- Integration of EEG and neuroimaging approaches in a comprehensive model is not well described.
- A specific measure of resilience is not included.

5. Environment:

Strengths

- The main research will be performed at the Minneapolis VA Health Care System and Center for Magnetic Resonance Research (CMRR) at the University of Minnesota. These institutions have excellent facilities to perform the proposed work at a high standard.

Weaknesses

- None noted.

Protections for Human Subjects:

Acceptable Risks and/or Adequate Protections

- Acceptable risks.

Data and Safety Monitoring Plan (Applicable for Clinical Trials Only):

Not Applicable (No Clinical Trials)

Inclusion of Women, Minorities and Children:

- Sex/Gender: Distribution justified scientifically
- Race/Ethnicity: Distribution justified scientifically
- For NIH-Defined Phase III trials, Plans for valid design and analysis: Not applicable
- Inclusion/Exclusion of Children under 18: Excluding ages <18; justified scientifically
- Children not eligible for military service.

Vertebrate Animals:
Not Applicable (No Vertebrate Animals)

Biohazards:
Not Applicable (No Biohazards)

Budget and Period of Support:
Recommend as Requested

CRITIQUE 3

Significance: 2
Investigator(s): 2
Innovation: 2
Approach: 2
Environment: 2

Overall Impact: This is a new submission from Dr. Melissa Polusny and Dr. Christopher Erbes, clinical psychologists from the University of Minnesota, addressing models of resilience in new military recruits of the US National Guard (NG) using basic training (BCT), as a stress exposure paradigm. The integrated team will collaborate to develop a comprehensive dynamic model of resilience using a multi-level perspective. Minor weaknesses include the lack of direct measures of psychological resilience and coping.

1. Significance:

Strengths

- The Public Health significance of the proposed research is high due to military services, during basic training or in war, is a stressor and can test one's resilience. With about 20% unable to complete basic training, it is important to understand features of resilience that can help selection and predict patterns of adaptation to Military service in the new recruits.
- The central scientific premise is that the ability to self-regulate underlies resilience and adaptation to the demands of basic training and serving in the NG. The hypotheses address the different factors involved in self-regulation of stress (the flexible regulation of attention, cognition, emotion, and behavior) as a key mechanism contributing to resilience in response to adversity/stressor exposure, which could be targeted for prevention and treatment using mind-body approaches.

Weaknesses

- None noted.

2. Investigator(s):

Strengths

- Overall the investigative group is excellent with appropriate experience and prior collaboration record. The Principal Investigators are clinical psychologists who have collaborated for 10 years and conducted federally funded research on risk and protective factors predictive of mental health and resilience following exposure to a range of traumatic experiences. Dr. Polusny has directed the Readiness and Resilience in National Guard Soldiers (RINGS) research lab at the Minneapolis VA Medical Center with longitudinal cohort studies with over 6,000 soldiers. She is a Principal Investigator on the longitudinal study of pain and pain management strategies among OEF/OIF Veterans funded by the NCCIH. Dr Erbes has studied social and environmental predictors and outcomes as they relate to distress, pathology, and resilience in the National Guard soldiers. He also studied the effect of MDBSR in PTSD. Other members of the study team (Arbisi, Sponheim, and Davenport) have collaborated with the Principal Investigators before. The team has sufficient and complementary expertise in traumatic stress, adversity, and resilience; longitudinal methods; advanced statistical modeling; neuroscience and imaging; experimental psychopathology; developmental psychology; and mind-body interventions.
- Excellent team of investigators with sufficient expertise.

Weaknesses

- None noted.

3. Innovation:

Strengths

- Innovation of the study lies in the integration of novel laboratory-based experimental paradigms for assessing dynamic self-regulatory processes within the context of a large, prospective, longitudinal study.
- The focus on self-regulatory processes in stress response as the basis of resilience is novel, especially when applied to the military recruits and basic service situation.
- The model of resilience is well thought out and comprehensive and includes the use of neural biomarkers of self-regulation, supported by the investigators prior research.

Weaknesses

- None noted.

4. Approach:

Strengths

- The two-phase approach will allow conducting a pilot study, planning and preparing for a longitudinal study of resilience. Phase 1 will make use of prior data sets made up of 4 waves of observation on over 3,000 NG soldiers and/or spouses before and after stressor exposure, and novel data collection from a pilot cohort of 100 NG recruits with survey and neurobehavioral assessment. Phase 2 will involve pre-BCT and 4 waves of post-BCT self-report data collection to characterize resilience vs. non-resilience trajectories and their predictors. Comprehensive

neurobehavioral assessments using MRI imaging will be investigated in 120 of new recruits who will complete laboratory tasks (Farmer's task, go/no-go task, dot probe task, performance monitoring task) before and after BCT, while undergoing MRI or EEG scans that will be related to resilient/non-resilient trajectories.

Weaknesses

- Lacks direct measures of psychological resilience and coping.

5. Environment:

Strengths

- Outstanding scientific environment with a track record in PTSD and resilience in the military.
- Strong institutional support of the infrastructure for the Principal Investigator and the project.

Weaknesses

- None noted.

Protections for Human Subjects:

Acceptable Risks and/or Adequate Protections

- Appropriate.

Data and Safety Monitoring Plan (Applicable for Clinical Trials Only):

- Acceptable.

Inclusion of Women, Minorities and Children:

- Sex/Gender: Distribution justified scientifically
- Race/Ethnicity: Distribution justified scientifically
- For NIH-Defined Phase III trials, Plans for valid design and analysis: Not applicable
- Inclusion/Exclusion of Children under 18: Excluding ages <18; justified scientifically

Vertebrate Animals:

Not Applicable (No Vertebrate Animals)

Biohazards:

Not Applicable (No Biohazards)

Budget and Period of Support:

Recommended budget modifications or possible overlap identified:

- Some of the salary support requests do not follow NIH cap rule.

CRITIQUE 4

Significance: 3

Investigator(s): 1
Innovation: 2
Approach: 4
Environment: 1

Overall Impact: The proposed project aims to study resilience in terms of self-regulatory processes in National Guard recruits in Minnesota. In Phase 1, using prior datasets, statistical algorithms will be developed for selecting participants with high and low probabilities of resilience. These datasets were not well described. The feasibility of recruitment and the feasibility and acceptability of neurobiological measures before and after basic training will be piloted. In Phase 2, online self-report assessments will be conducted before basic training and at four points after. Resilient and non-resilient trajectories will be defined. One hundred and twenty recruits will complete laboratory tests on self-regulatory processes before and after basic training by undergoing EEG and MRI scans. Analyses will determine whether these biologically oriented tasks can discriminate between the trajectories based on self-report data. This project is innovative in comparing self-report to more biologically-based assessments. Due to the selection process for National Guard recruits and issues of stigma and appearing weak, it is unclear whether the team will be able to adequately detect, recruit, and retain the sample – especially for the non-resilient group.

1. Significance:

Strengths

- This study address an important problem of how to assess for resilience, using both self-report and more biologically-based measures.
- If the applicant achieves the aims of the application, it will advance scientific knowledge and clinical practice.
- Their findings could inform new and potentially more effective programs for enhancing resilience from either preventive or treatment perspectives.

Weaknesses

- The prior National Guard datasets used to establish predictive algorithms were not well described.
- The major concern is regarding the team's ability to recruit and retain enough National Guard recruits in this study. The team mentioned their prior success in this area but it was not clear whether the prior studies were as time intensive and invasive.

2. Investigator(s):

Strengths

- A very strong interdisciplinary team with a history of working together effectively and with the population of focus.

Weaknesses

- None identified.

3. Innovation:

Strengths

- The laboratory-based paradigms for assessing self-regulatory processes are novel.

Weaknesses

- The team is noncommittal regarding the neurobehavioral approaches for assessing self-regulation and say that after a literature review and pilot testing the plan as presented in this proposal could be modified.

4. Approach:

Strengths

- The proposed project builds on decades of high quality research.

Weaknesses

- A concern that was not addressed by the team is the issue of selection factors. Given that National Guard recruits are screened, it is questionable whether there will be enough variation in terms of non-resilient recruits.
- Those who are more resilient might be more likely to agree to participate or continue in this study. There could be differential attrition in the non-resilient group.
- It is unclear whether deployment or drop-out of the National Guard could affect follow-up assessments. The application does not state whether they will continue to follow-up those recruits who separate from the National Guard.
- Given the challenges in military personnel with stigma and appearing weak there may not be adequate variation in the data to distinguish between resilient and non-resilient recruits. Given this limitation with reporting bias, the laboratory test data might provide better prediction than self-report assessments.
- Linkage to other records, on National Guard disciplinary records, promotion and career advancement, etc., could provide added information on resilience for minimal costs.
- Blood will be collected and could be used to inform the models with blood-based biomarkers.

5. Environment:

Strengths

- Uniformly strong.

Weaknesses

- No weaknesses.

Protections for Human Subjects:

Acceptable Risks and/or Adequate Protections

- Appropriate.

Data and Safety Monitoring Plan (Applicable for Clinical Trials Only):

Not Applicable (No Clinical Trials)

Inclusion of Women, Minorities and Children:

- Sex/Gender: Distribution justified scientifically
- Race/Ethnicity: Distribution justified scientifically
- For NIH-Defined Phase III trials, Plans for valid design and analysis: Not applicable
- Inclusion/Exclusion of Children under 18: Excluding ages <18; justified scientifically

Vertebrate Animals:

Not Applicable (No Vertebrate Animals)

Biohazards:

Not Applicable (No Biohazards)

Resource Sharing Plans:

Acceptable

Budget and Period of Support:

Recommend as Requested

THE FOLLOWING SECTIONS WERE PREPARED BY THE SCIENTIFIC REVIEW OFFICER TO SUMMARIZE THE OUTCOME OF DISCUSSIONS OF THE REVIEW COMMITTEE, OR REVIEWERS' WRITTEN CRITIQUES, ON THE FOLLOWING ISSUES:

PROTECTION OF HUMAN SUBJECTS: ACCEPTABLE

INCLUSION OF WOMEN PLAN: ACCEPTABLE

INCLUSION OF MINORITIES PLAN: ACCEPTABLE

INCLUSION OF CHILDREN PLAN: ACCEPTABLE

COMMITTEE BUDGET RECOMMENDATIONS: The budget was recommended as requested.

Footnotes for 1 UG3 AT009651-01; PI Name: POLUSNY, MELISSA A.

NIH has modified its policy regarding the receipt of resubmissions (amended applications). See Guide Notice NOT-OD-14-074 at http://grants.nih.gov/grants/guide/notice-files/NOT-OD-14-074.html. The impact/priority score is calculated after discussion of an application by averaging the overall scores (1-9) given by all voting reviewers on the committee and multiplying by 10. The criterion scores are submitted prior to the meeting by the individual reviewers assigned to an application, and are not discussed specifically at the review meeting or calculated into the overall impact score. Some applications also receive a percentile ranking. For details on the review process, see http://grants.nih.gov/grants/peer_review_process.htm#scoring.

MEETING ROSTER

The roster for this review meeting is displayed as an aggregated roster that includes reviewers from multiple CSR Special Emphasis Panels of the Risk, Prevention and Health Behavior Intergrated Review Group
for the 2017/05 council round.
This roster for CSR is available at:
http://public.era.nih.gov/pubroster/Reports?DOCTYPE=SEP&DESFORMAT=PDF&AGENDA_SEQ_NUM_P=318632
