# Supplementary material for: Adaptation in Young Military Recruits: Protocol for the Advancing Research on Mechanisms of Resilience (ARMOR) Prospective Longitudinal Study
Source: JMIR Res Protoc. 2023 Oct 4;12:e51235. doi: 10.2196/51235 (PMC10585449; doi:10.2196/51235)
Supplement: Multimedia Appendix 2 [file resprot_v12i1e51235_app2.pdf]

Overview

The overarching goal of the proposed supplement is to rapidly improve our understanding of resilience processes related to the economic, social, and personal impacts of the COVID-19 pandemic[1], including adherence to COVID-19 containment/ mitigation efforts, and their downstream mental health outcomes in National Guard soldiers. We will capitalize on an ongoing longitudinal cohort study of 1,200 National Guard new recruits residing across the state of Minnesota (5UH3AT009651-03; PIs: Polusny & Erbes). In the UH3 parent project, all recruits are prospectively assessed prior to basic combat training (BCT) and followed up at four time points post-BCT (at 6-month intervals). When Minnesota announced its stay-at-home order in March 2020, we had completed baseline data collection with 755 of 1,200 participants. We anticipate resuming baseline data collection for the UH3 parent grant in July-August 2020 allowing for a natural experiment[2]. Our objectives for the proposed supplement are to: ***1) determine whether pre-pandemic indicators of self-regulation, pre- and during-pandemic social and organizational support (resilience factors) attenuate downstream mental health responses to COVID-19-related stressors; 2) evaluate the role of National Guard BCT, self-regulation, social and organization support in predicting adherence to COVID-19 containment efforts; and 3) evaluate the role of BCT as a potential resilience factor attenuating the link between COVID-19-related stressors and downstream mental health outcomes.***

Statement on NCCIH UH3 Parent Grant

The overarching goal of the UH3 parent grant is "to develop an integrative model that elucidates mechanisms and processes of resilience using a multi-level perspective." It was explicitly designed to examine how "personality traits and social processes are promotive (main effect) or protective (interactive with stressors) in adults and how these processes interact with self-regulation to promote resilience." This supplement will capture reliable, valid, and time-critical data to characterize participants' exposure and responses to a range of COVID-19-related stressors across multiple timepoints during the pandemic. By characterizing this novel and unanticipated stressor and studying potential resilience factors related to it, this supplement will greatly increase the potential impact of the parent study[3].

Significance of the Problem and Knowledge to be Gained

While there is no single accepted definition, we define resilience as the capacity for positive adaptation in the context of significant stress or adversity[4,5]. Resilience is a dynamic, multidimensional process, not a static trait or simply lack of pathology at a single time point[6]. At the broadest level, adaptive responding involves low levels of psychopathology accompanied by healthy social (relationship, academic, occupational, community) functioning. Military service provides a promising context for the study of resilience. Even during peace time, service members are confronted with multiple stressors and challenges unique to military life. All military recruits are required to complete a course of **basic combat training (BCT)**, a well-defined uniform period of intense stress that provides an ideal context for studying resilience. Although it is designed to enhance fortitude and toughness (aspects of resilience), BCT is an intensive 10-week training period involving dramatic changes in living environment, mental challenges, and extreme physical demands[7]. Military training stressors include prolonged separation/isolation from family/friends, dramatic changes in living environment, prolonged simulated combat exposure, extreme physical demands, and mental challenges[7–10]. While most military recruits successfully adapt to these challenges, nearly 20% of National Guard military recruits fail to complete BCT[10]. Mental health and conduct problems are among the most common reasons for early attrition of military recruits[11,12]. Thus, BCT represents both a potential stress inoculating intervention designed to facilitate resilience and a potent stressor requiring intact resilience processes to overcome.

The coronavirus (COVID-19) pandemic poses profound challenges for the mental health and well-being of the population[13–15], including National Guard recruits. First, with exponential increases in COVID-19 cases and deaths, recruits may themselves become ill or fear infection. Second, recruits may experience a host of social, occupational, financial, and familial stressors confronting the population as a whole secondary to stay-at-home/lockdown orders and adherence to COVID-19 containment efforts (e.g., increased social isolation, loneliness, sedentary lifestyle). Third, recruits may be exposed to additional COVID-19-related stressors as a result of being mobilized in support of local or national emergency response efforts. For example, Minnesota Army National Guard members, from whom ARMOR participants are drawn, have been mobilized to aid with COVID-19 testing in long-term care facilities. One means of successful adaptation to the COVID-19 pandemic is adhering to containment efforts[1] (e.g., quarantine, stay-in-place, physical distancing and isolation) designed to prevent the spread of the disease. We are aware of no studies that have investigated adherence to COVID-19 prevention measures in this important population.

The ARMOR (UH3 parent) study builds on over four decades of developmental research pointing to three fundamental factors in resilience[16–19]. These include: (1) positive affectivity, or the ability to experience enduring and appropriate positive affect, (2) supportive relationships that buffer individuals from the effects of adversity, and (3) executive functioning and other higher-order cognitive skills needed for self-regulation in the face of stress[20]. Self-regulation of attention, cognition, and behavior is associated with responding effectively to adversity and is protective against the deleterious effects of stress[6,21]. Predisposing individual differences factors (e.g., biologically-based personality traits) and social contextual factors can influence or interact with these self-regulation processes to influence the effects of stressor exposure/adversity on outcomes[22]. Evidence suggests these factors continue to exert influence on resilience as youth transition to emerging adulthood[23–25]. A substantial body of research across the lifespan shows that deficits in self-regulation are associated with greater risk for a range of problems including psychopathology[26–28], substance abuse[29,30], sexual risk taking[31], and poor physical health[32,33]. While researchers have documented the impact of pandemics on mental health[34], a dearth of studies have examined resilience processes such as self-regulation and social supports as potential moderators of those impacts. We will capitalize on the unique timing and nature of the ongoing ARMOR study to address this gap. We have three broad hypotheses based on the ARMOR overarching model: *1) We hypothesize that self-regulation, social and organizational support will interact with (reduce the effect of) COVID-19-related stressors in predicting mental health outcomes over time; 2) We hypothesize that adherence to COVID-19 containment/mitigation efforts will be predicted by baseline measures of self-regulation and ongoing measures of social and organizational support. We also hypothesize that those participants who have completed BCT will show higher rates of adherence to COVID-19 containment efforts; 3) We hypothesize that participants who competed BCT prior to the onset of COVID-19 will show reduced relationships between COVID-19-related stressors and mental health outcomes at follow-up.*

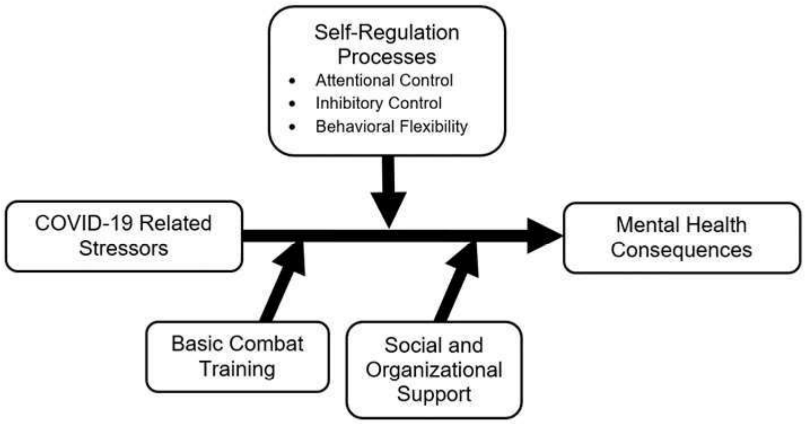

Figure 1. Conceptual model of resilience: self-regulation, support & basic combat training

Advantages of the Proposed Supplemental Application

A key challenge of research in the context of disasters or mass traumas (i.e., pandemics) is that often it is unclear (without assessment prior to exposure) whether observed or reported differences following the event are related to exposure[35]. The proposed supplement takes advantage of a rare event (global pandemic occurring during an ongoing longitudinal study of resilience) among a population likely to be vulnerable to exposure (National Guard personnel mobilized to support the fight against COVID-19). The supplement capitalizes on prospective data from our ongoing longitudinal study of National Guard recruits in order to study the severity and predictors of COVID-related behaviors and mental health outcomes. ARMOR was developed specifically to study mental health stress responses and resilience in this population. Participants are already reporting on a range of military and civilian stressors and important resilience factors (such as self-regulation and social support) and risk factors (such as vulnerable personality traits) and are reporting on potential mental health outcomes over a two-year span. Furthermore, over half of ARMOR participants (N=755) have already been recruited and assessed prior to the pandemic outbreak. As such, we will be able to prospectively evaluate the role of resilience and risk factors assessed pre-pandemic in moderating during and post-pandemic responses. An additional 425 ARMOR participants have yet to be enrolled and will complete baseline measures following the pandemic outbreak. This provides a rare opportunity to conduct a “natural experiment[2]” in which recruits who have completed the stressful but potentially resilience enhancing BCT prior to the pandemic can be compared to those who have not, allowing an evaluation of the role of BCT in moderating (attenuating) COVID-19-related stress responses. Finally, we will be able to contrast the protective roles of self-regulation and social support measured during the pandemic (i.e., in the 425 future participants) to those same factors that were measures prior to the pandemic.

DATA COLLECTION FOR THE UH3 PARENT

ARMOR is a 4-year observational, mixed methods longitudinal study with a new cohort (N=1200) of recently enlisted Army National Guard soldiers to develop an integrative multi-level model of resilience. The study design is shown in Figure 2a and 2b. These figures show the Survey Component, in which participants are assessed using computerized self-administered questionnaires and neurocognitive tests at baseline, prior to shipping to BCT. All participants are followed for four additional waves beginning 2-weeks post-BCT, with surveys administered at 6-month intervals. Self-report measures assess personality traits (baseline only), mental health symptoms, social functioning, social support, trait resilience, and self-regulation processes (at all timepoints). Computerized neurocognitive tests (baseline only) assess cognitive capacity and executive function. Primary outcomes are resilient and non-resilient trajectories of self-reported internalizing symptoms, externalizing symptoms, social functioning, and global adaptive functioning. Secondary outcomes are self-reported and performance-based measures of self-regulatory processes as well as real-world markers of functioning extracted from administrative military records (e.g., premature attrition from military service, demotion). To maximize retention in the parent study, we adopted Scott's evidence-based four-phase (Engagement, Verification, Maintenance, and Confirmation; EVMC[36]) model. Studies using the EVMC model have achieved follow-up rates of 80% or better[37–39]. In the laboratory component, (not pictured in Figure 2) a subset of 120 participants, selected to be at high or low risk for poor versus resilient outcomes, complete a laboratory visit pre- and post-BCT. Laboratory procedures include a structured clinical interview for DSM-5 disorders, neurobehavioral tasks (Dot Probe Task, Performance Monitoring Task, and Go/No-Go Task) performed during an EEG assessment session, and the Farmer's Paradigm Task (baseline only) performed while in a functional MRI scan. Saliva and blood are also collected and stored for future research.

PROPOSED ACTIVITIES FOR THE ADMINISTRATIVE SUPPLEMENT

This supplement proposes to collect two additional COVID-19 focused waves of survey data over the next two years to enhance and further the activities of the UH3 parent grant described above. **All eligible new recruits enrolled in the ongoing UH3 parent study will be recruited to participate in the supplemental COVID-19 waves (hereafter referred to as the CO).** CO waves will be collected using our university's secure Qualtrics online survey platform following the same procedures used in the UH3 parent study. Broadly, CO surveys will assess: 1) participants' (self and loved ones) experiences with COVID-19; 2) exposure to COVID-19-related stressors; 3) adherence to containment/mitigation efforts; 4) use of social supports and self-regulation; and 5) downstream mental health outcomes. **Table 1** shows measures to be administered at each CO wave. Measurement consideration was guided by discussions with fellow OppNet Resilience UH3 PIs to ensure harmonization for future collaborative work. COVID-19 focused measures were selected from the PhenX Toolkit and NIH Repository of COVID-19 Research Tools[40] (see details below under Measures). We will index COVID-19-related stress using a widely used measure of perceived stress[41]. The 3-item Loneliness Scale[42] will be used to assess impact of social isolation, and a brief measure will index impact on physical activity[43]. In addition, we will modify the instruction sets of

Table 1. Constructs and measures assessed in CO surveys

Construct	Measure	# items
Adherence to COVID-19 Containment Efforts	COvid-19 Participant Experience (COPE): Social Distancing	10
	Epidemic-Pandemic Impacts Inventory (EPII): Physical Distancing and Quarantine; Infection History	15
COVID-19 Related Stressors: Economic, Social, and Personal Impacts	EPII: Work & Employment; Education & Training; Home Life; Social Activities; Economic; Emotional Health & Well-Being; Physical Health Problems; Positive Change	75
	Loneliness Scale-3	3
	Perceived Stress Scale (PSS)	10
	International Physical Activity Questionnaire (IPAQ)	8
Substance Use	COPE Consortium Tool/Lifestyle Module	2
	Alcohol Use Disorder Test (AUDIT)	10
	Drug Abuse Screening Test (DAST)	1-10
Mental Health	PROMIS-Depression 4	4
	PROMIS-Anxiety 4	4
	PROMIS-Anger 5	5
	Primary Care PTSD Screen DSM-5	4
	Patient Health Questionnaire-15: Somatization (PHQ-15)	15
Social Functioning	Utrecht Work Engagement Scale	9
	Endicott Work Productivity Scale	15
	Conflict Tactics Scale-2 (CTS-2-COVID-19)	2
	Couple Satisfaction Inventory (CSI-COVID-19)	4
Social Support	Emotional Support – Short Form 4a	4
	Informational Support – Short Form 4a	4
	Instrumental Support – Short Form 4	4
Self-Regulation	Difficulties in Emotional Regulation Scale (DERS-16)	16
	Brief Multidimensional Experiential Avoidance Questionnaire (MEAQ-30)	30

measures of COVID-19-related social supports (PROMIS Social Support-short scales[44]) and social functioning (Utrecht Work Engagement Scale[45], Endicott Work Productivity Scale[46], Conflict Tactics Scale-2-COVID-19[47], and Couples Satisfaction Inventory-CSI-COVID-19[48]) to cue participants to the pandemic. Mental health and substance use will also be assessed using the same measures as those administered in the UH3 parent study. When possible, we selected measures parallel to those administered in the ARMOR baseline survey (AR0). This will allow us to observe changes in outcomes measured prospectively over the course of the pandemic as well as examine how the unfolding of COVID-19-related impacts on subsequent outcomes.

Timing of COVID-19 Survey Waves

As the ARMOR study was in active recruitment prior to the COVID-19 pandemic, our design allows the assessment of three natural cohorts within the ARMOR sample. About half the ARMOR sample was recruited/completed baseline data collection **before the COVID-19 pandemic** (Cohorts 1 and 2), and remaining participants will be recruited **after/during the pandemic.** Cohort 1 is composed of participants who had completed the baseline survey (AR0), BCT, and one follow-up wave (AR1) prior to the onset of the pandemic. Cohort 2 is composed of participants who had completed AR0 prior to the pandemic, but not BCT. Some of Cohort 2 had shipped for BCT prior to the pandemic, while others had not yet been shipped to BCT. Cohort 3 will be made up of participants who will enroll in the study during the COVID-19 pandemic. For Cohort 3, AR0, BCT, and all subsequent waves will occur after the onset of the current COVID-19 crisis. The timing of the two additional COVID-19 survey waves will vary by cohort. As shown in **Figure 2a**, Cohort 1 will receive CO1 prior to AR2 and CO2 prior to AR3, Additional sets of items will be collected with AR3 and AR4 (termed CO3 and CO4 in Figure 2a). In contrast, for Cohort 2 CO1 will occur either before BCT (prior to AR1) or immediately after (just after AR1) and CO2 will occur prior to AR2. In Cohort 3, CO1 will occur prior to BCT, and CO2 will occur following BCT (just after AR1). For Cohorts 2 and 3 additional COVID-19-related items will be collected in AR2, AR3, and AR4 (CO3, CO4, and CO5 in Figure 2b). As Cohort 1 will not receive CO5, that wave will be considered missing by design in the analyses (see below). The timing between CO1 and CO2 will typically be 4 months, though this may be slightly longer if a participant has a delayed return from BCT.

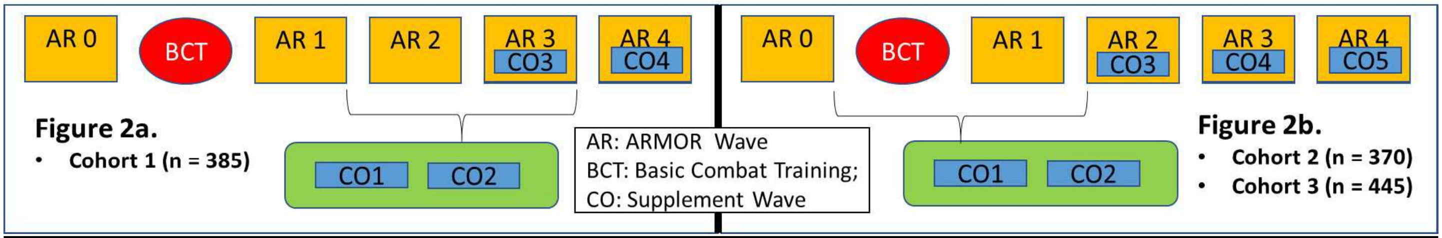

Integration of COVID-19 Survey Waves with the Laboratory Component

A strength of the UH3 parent study is it's use of behavioral and physiological assessments to help examine resilience processes related to self-regulation. However, as the laboratory portion is completed by only 120 participants who will be distributed across Cohorts 1, 2, and 3, we may not be adequately powered to test hypotheses related to task performance, brain activity, and brain morphology predicting COVID-19-related stress responses and behaviors. We do plan to conduct exploratory analyses in this regard.

COVID-19 Focused Measures

Epidemic-Pandemic Impacts Inventory (EPII)[49] is a 92-item measure designed to assess participants' experiences with COVID-19 pandemic and its impact on various economic, social, and personal domains. Respondents are asked, "Since the coronavirus disease pandemic began, what has changed for you or your family?" Respondent indicate whether they or another person (people) in their home were impacted by a series of items that are organized into personal and family life domains. Items from the EPII *Infection History* domain will be used to characterize participants' self-reported experiences with infection and illness related to COVID-19. Exposure to a wide range of COVID-19-related stressors will be assessed using the following EPII domains: *Work and Employment, Education and Training, Home Life, Social Activities, Economic, Emotional Health and Well-Being, Physical Health Problems, and Positive Change.* The International Physical Activity Questionnaire-Short Form (IPAQ-SF)[43] is a 7-item scale developed to assess the intensity of physical activity and sitting that people do as a part of their daily lives. Finally, we will assess exposure to National Guard deployments (i.e., being called to duty) for COVID-19 or other crisis duties. All of Us Research Program COvid-19 Participant Experience (COPE) Social Distancing[50] is a 10-item questionnaire that measures participants reports of adherence to recommended public health guidelines designed to contain the spread of COVID-19

(e.g., washing hands frequently, avoiding touching face, covering coughs, avoiding frequently touched surfaces in public places, staying at home, social distancing, and avoiding close contact with persons in vulnerable risk groups). Finally, items from the EPII[49] *Physical Distancing and Quarantine* domain will be used to assess adherence to containment efforts (e.g., isolation or quarantined efforts).

Analysis plan

Figure 3 illustrates the waves of data collected for the UH3 parent grant (AR0 through AR4), waves to be collected as part of this supplement (CO1 to CO5), and the exposure to basic combat training (BCT) and the COVID-19 pandemic (COVEXP) at each timepoint for each of our naturally occurring cohorts. COVEXP 0 waves are shown in black, while COVEXP 1 waves are shown in red. X represents potential resilience or risk factors (e.g., self-regulation, social support), Y represents outcomes (e.g., mental health or social functioning) and CRV represents COVID-19-related stressors and behaviors. This demonstrates a naturally occurring repeated measures factorial block design with three blocks (cohorts) and two within-subjects factors: BCT (0,1) and COVEXP (0, 1), as shown in the Within Subjects Factors panel of Figure 3. Figure 3 illustrates that for ARMOR, one wave is pre-BCT (BCT 0) while four are post-BCT (BCT 1) while for CO waves, the distribution from pre- to post-BCT varies. Note that while all participants in Cohort 2 complete AR0 under COVEXP = 0, some will complete BCT after CO1 (Cohort 2a) while others will complete BCT before CO1 (cohort 2b). Also, CO1 is missing-by-design for Cohort 1. Analyses will preliminary estimate the main effects of BCT and COVEXP on outcomes (Y) but, more importantly, their interactive effects in predicting Y and CRV.

Figure 3. ARMOR and Supplement assessment and study design.

		ARMOR Waves					Supplement Waves					Within Subject Factors	
Cohort	n	BCT=0	BCT=1				BCT = 0	BCT = 1				BCT = 0	BCT = 1
		AR0	AR1	AR2	AR3	AR4	CO1	CO2	CO3	CO4	CO5		
1	385	Y0X0	Y1X1	Y2X2	Y3X3	Y4X4	NA	CRV2	CRV3	CRV4	CRV5	BCT0, COVEXP 0	BCT1, COVEXP 0
2a/b	370	Y0X0	Y1X1	Y2X2	Y3X3	Y4X4	CRV1 (2a)	CRV2 (2a) CRV1&2 (2b)	CRV3	CRV4	CRV5	BCT0, COVEXP 0	BCT1, COVEXP 1
3	445	Y0X0	Y1X1	Y2X2	Y3X3	Y4X4	CRV1	CRV2	CRV3	CRV4	CRV5	BCT0, COVEXP 1	BCT1, COVEXP 1

Notes. X = risk/resilience variables (self-regulation, social support), Y = outcomes (mental health, social functioning), CRV = COVID Related Variables (COVID exposure, related stressors, containment behaviors). Red text indicates data collected post-COVID pandemic onset, black indicates pre-COVID pandemic data. Blue background represents post-BCT data collection, gray background represents pre-BCT data collection. CO waves after the first two (e.g., CO4 and CO5) will be collected simultaneously with ARMOR waves.

Preliminary Analyses. Initially we will test various hypotheses of direct and indirect effects of BCT and COVEXP on outcomes (Y), resilience factors (X) and CRVs using simple univariate analysis of variance models for repeated measure factorial block design, without adjusting for any possible confounders, on both complete cases and multiply imputed data sets. We will then augment these analyses with three sets of MANOVAs that can account for the interdependencies within the set of Y, X, and CRV variables. These MANOVAs will help us to model the possible differential impact of race/ethnicity and gender and identify sub-cohorts of various vulnerabilities. Note that MANOVAs makes it possible to run Profile Analysis by running different contrasts. We will run three separate sets of repeated measure MANOVAs, all with the same set of main effects of BCT, COVEXP, and their interaction, after adjusting for the block (cohort) effect. The first set, will model the outcomes (Ys) jointly, the second, protective factors jointly (Xs), and finally, our various measures of CRVs to see if they vary by these factors. Identifying confounders of BCT and COVID exposure on these three sets and understanding their implications is necessary. To this end, after investigating the balance question of the collected concomitant variables, we will augment the previous unadjusted analyses with the corresponding multivariate analysis of covariances using the imbalance confounders.

Objective 1 Analysis. *We hypothesize that self-regulation, organizational support, and social support (Xs) will interact with (reduce the effect of) COVID-19-related stressors (CRV) in predicting mental health outcomes (Ys).* The outcome variables as well as COVID-19-related stressors are continuous variables. The moderating variables would be various indices of self-regulation and social supports (Xs). The outcomes would include mental health including anxiety (ANX), depression (DEP), somatization (SOM) and alcohol misuse (ETOH) as well as indices of social functioning (e.g., PROMIS Social Role Participation and Satisfaction).

We will use a series of generalized linear mixed models for the mean of these outcomes repeated measures over five waves. The Baseline Outcome, Cohort, time, BCT and COVEXP factors, and time-varying

moderators and COVID-related stressor exposure and their interaction terms will be the predictors of this model. Initially, a compound symmetric and auto-regressive covariance structure will be used to capture the dependence between the repeated outcome via a random-intercept model with various tests on the two-way interactions in the model. Finally, using fit indices (such as BIC we will improve the fit by modeling the time trend with higher degree polynomials (here it can go up to 4 without over fitting). The interaction between COVID stressors and Protective factors (X by CVR), if significant, will give an idea of how these protective factors change the effect COVID stressors on the outcome (and vice versa, how these stressors modulate the effect of protective factor). We may then use this final model to study some interesting contrasts like pre-COVEXP vs post COVEXP, and pre BCT vs post BCT on outcomes (Y). Fortunately, these contrasts are estimable. Note that we will also augment this set of analyses with adjustments due to confounders detected in the preliminary MANCOVAs.

Objective 2 Analysis. *We hypothesize that adherence to COVID-19 containment/mitigation efforts at CO1 and all CO follow-up timepoints will be predicted by AR0 measures of self-regulation and CO1 measures of social and organizational support. We also hypothesize that those participants who have completed BCT will show higher rates of adherence to COVID-19 containment efforts.* Adherence is a time varying behavior that will be measured either as a summary measure of adherence in different domains (social distancing, hand washing, etc.). This will be calculated via the first principal component from the items (Likert-type scale) that report on this behavior at each time point. This sequence of five first PCAs will be used as our time-varying dependent variable. Our model will have the participant baseline self-regulation and social support, and his mental health status at the baseline, BCT (BCT=0,1), COVEXP (COVEXP = 0,1) as fixed effects. This simple mixed model (with just random intercept) will give us an initial understanding of how these protective factors (after adjusted for the mental health status at baseline) influence the adherence. The interaction between BCT factor and these protective factors will give us some insight in possible modifying effect of BCT. However, the next step is to add all the five measurements and mental health progression of subjects (and BCT, COVEXP, and the time trend) and their interaction on adherence adjusting for COVID-induced repeated stress measurements. As before, the generalized mixed modelling of the five dependent outcomes will be replicated with adjustment for confounding covariates. The dimensionality of confounders will be reduced via PCA if required.

Objective 3 Analyses. *We hypothesize that participants who competed BCT prior to the onset of COVID-19 (those in Cohort 1) will show reduced relationships between COVID-19-related stressors and mental health outcomes at follow-up timepoints*. Figure 3 shows that our cohorts are collected at different time points and under different conditions regarding BCT and COVEXP. Cohort 1 are participants who had gone through BCT prior to COVID-era, while Cohort 3 had the BCT training and their pre-BCT measurement were taken post COVID exposure. Cohort 2 is a mixture population. We will examine whether the effect of COVID-induced stressors is the same between Cohorts, especially Cohort 1 and 3. This can be done in at least two ways. 1) use the same generalized linear mixed modeling of outcomes in term previously mentioned fixed adjusting terms and COVID stressors as a main predictor and compare the coefficients of BCT by COVID stressor across the two cohorts (Cohort 1 and Cohort 3). Since they are independent samples, a simple z-test(t-test) will show if there is differential modifying contribution of BCT on the effect of these stressors on the outcomes between the two cohorts. The direction of the effect can be conjectured from the sign of change. 2) Combing the above two models into a grand mixed model with three-way interaction of *Block by COVID stressor by BCT* (with all the lower order interaction). Significance of these three-way interaction will provide some quantitative indication of varying modifying effect of BCT on mental health stress responses.

Missing Data and Limitations. All the above work will be done on available data as well as multiply imputed data. If missingness is not completely at random, we will use multiple imputation techniques to judge the robustness of the analysis using complete cases, as suggested by Little and Rubin (2003). Based on the measured covariates, conditional probabilities or distributions for the possible values of the missing data will be constructed using generalized linear models in a sequential manner at each time points condition on the past history. Subsequent analysis will sample randomly from these estimated distributions and implement the analyses described above. These imputation analyses will be repeated in numerous replications and measures of association will be constructed using averages of the estimates and p-values obtained in the individual replications. To account for the extra variation introduced into estimates by imputation, we will use the R packages "MICE," "Amelia" or "mi". Note that we have not performed any sample size calculation, as this grant is supplement to our UH3 parent study with approximately 1200 subjects (see Figure 3) above. In other words, the sample size is fixed. However, we plan to report post hoc powers for the main tests pertinent to each objective.

References Cited

1. Brooks SK, Webster RK, Smith LE, et al. The psychological impact of quarantine and how to reduce it: rapid review of the evidence. *Lancet*. 2020;395(10227):912-920.
2. Patrick SL, Cormier HC. Are our lives the experiment? COVID-19 lessons during a chaotic natural experiment–A commentary. *Heal Behav Policy Rev*. 2020;7(2):165-169.
3. Holmes EA, O'Connor RC, Perry VH, et al. Multidisciplinary research priorities for the COVID-19 pandemic: a call for action for mental health science.*The Lancet Psychiatry*. 2020;April 15.
4. Southwick SM, Bonanno GA, Masten AS, Panter-Brick C, Yehuda R. Resilience definitions, theory, and challenges: Interdisciplinary perspectives. *Eur J Psychotraumatol*. 2014;5.
5. Fletcher D, Sarkar M. Psychological resilience: A review and critique of definitions, concepts, and theory. *Eur Psychol*. 2013;18:12-23.
6. Masten AS. Regulatory processes, risk, and resilience in adolescent development. *Ann N Y Acad Sci*. 2004;1094:28-39.
7. Smith BN, Vaughn RA, Vogt D, King DW, King LA, Shipherd JC. Main and interactive effects of social support in predicting mental health symptoms in men and women following military stressor exposure. *Anxiety, Stress Coping*. 2013;26(1):52-69.
8. Martin PD, Williamson DA, Alfonso AJ, Ryan DH. Psychological adjustment during Army Basic Training. *Mil Med*. 2006;171(2):157-160.
9. Lieberman HR, Karl JP, McClung JP, Williams KW, Cable S. Improved mood state and absence of sex differences in response to the stress of Army Basic Combat Training. *Appl Psychol Heal Well-Being*. 2016;8(3):351-363.
10. Williams A, Hagerty BM, Yousha SM, Horrocks J, Hoyle KS, Liu D. Psychosocial effects of the boot strap intervention in Navy recruits. *Mil Med*. 2004;169(10):814-820.
11. Booth-Kewley S, Highfill-Mcroy RM, Larson GE, Garland CF. Psychosocial predictors of military misconduct. *J Nerv Ment Dis*. 2010;198(2):91-98.
12. Hoge CW, Auchterlonie JL, Milliken CS. Mental health problems, use of mental health services, and attrition from military service after returning from deployment to Iraq or Afghanistan. *JAMA*. 2006;295(9):1023-1032.
13. Pfefferbaum B, North CS. Mental health and the Covid-19 pandemic. *N Engl J Med*. 2020;Apr 13.
14. Torales J, O'Higgins M, Castaldelli-Maia JM, Ventriglio A. The outbreak of COVID-19 coronavirus and its impact on global mental health. *Int J Soc Psychiatry2*. 20AD;Mar 31.
15. Qiu J, Shen B, Zhao M, Wang Z, Xie B, Xu Y. A nationwide survey of psychological distress among Chinese people in the COVID-19 epidemic: implications and policy recommendations. *Gen Psychiatry*. 2020;33(2).
16. Masten AS, Cicchetti D. Risk and resilience in development and psychopathology: The legacy of Norman Garmezy. *Dev Psychopathol*. 2012;24(2):333-334.
17. Garmezy N, Ruttern M. Acute reactions to stress. In: Rutter M, Hersov L, eds. *Child and Adolescent Psychiatry: Modern Approaches*. Oxford: Blackwell Scientific Publications; 1985:152-176.
18. Masten AS, Cicchetti D. Resilience in development: Progress and transformation. In: Cicchetti D, ed. *Developmental Psychopathology Volume 4: Risk, Resilience, and Intervention.* 3rd ed. Wiley; 2016:271-333.
19. Masten AS. Ordinary magic: Resilience processes in development. *Am Psychol*. 2001;56(3):227-238.
20. Busso DS. Neurobiological processes of risk and resilience in adolescence: Implications for policy and prevention science. *Mind, Brain, Educ*. 2014;8(1):34-43.
21. Gross JJ. Emotion regulation: Affective, cognitive, and social consequences. *Psychophysiology*. 2002;39(3):281-291.
22. Wachs TD. Contributions of temperament to buffering and sensitization processes in children's development. *Ann N Y Acad Sci*. 2006;1094:28-39.
23. Obradović J, Burt KB, Masten AS. Pathways of adaptation from adolescence to young adulthood: Antecedents and correlates. *Ann N Y Acad Sci*. 2006;1094:340-344.
24. Masten AS, Burt KB, Roisman GI, Obradović J, Long JD, Tellegen A. Resources and resilience in the transition to adulthood: Continuity and change. *Dev Psychopathol*. 2004;16(4):1071-1094.
25. Burt KB, Paysnick AA. Resilience in the transition to adulthood. *Dev Psychopathol*. 2012;24(2):493-505.

26. Aldao A, Nolen-Hoeksema S, Schweizer S. Emotion-regulation strategies across psychopathology: A meta-analytic review. *Clin Psychol Rev*. 2010;30(2):217-237.
27. Beauchaine TP, Thayer JF. Heart rate variability as a transdiagnostic biomarker of psychopathology. *Int J Psychophysiol*. 2015;98(2):338-350.
28. Beauchaine TP, McNulty T. Comorbidities and continuities as ontogenic processes: Toward a developmental spectrum model of externalizing psychopathology. *Dev Psychopathol*. 2013;25(4):1505-1528.
29. Ersche KD, Williams GB, Robbins TW, Bullmore ET. Meta-analysis of structural brain abnormalities associated with stimulant drug dependence and neuroimaging of addiction vulnerability and resilience. *Curr Opin Neurobiol*. 2013;23(4):615-624.
30. Stanis JJ, Andersen SL. Reducing substance use during adolescence: A translational framework for prevention. *Psychopharmacology (Berl)*. 2014;231(8):1437-1453.
31. Quinn PD, Fromme K. Self-regulation as a protective factor against risky drinking and sexual behavior. *Psychol Addict Behav*. 2010;24(3):376-385.
32. Buckley J, Cohen JD, Kramer AF, McAuley E, Mullen SP. Cognitive control in the self-regulation of physical activity and sedentary behavior. *Front Hum Neurosci*. 2014;8:747.
33. Teixeira PJ, Carraça E V., Marques MM, et al. Successful behavior change in obesity interventions in adults: A systematic review of self-regulation mediators. *BMC Med*. 2015;13:84.
34. Restubug SLD, Ocampo ACG, Wang L. Taking control amidst the chaos: Emotion regulation during the COVID-19 pandemic. *J Vocat Behav*. 2020;119(103440).
35. Masten AS, Narayan AJ. Child development in the context of disaster, war, and terrorism: Pathways of risk and resilience. *Annu Rev Psychol*. 2012;63:227-257.
36. Scott CK, Sonis J, Creamer M, Dennis ML. Maximizing follow-up in longitudinal studies of traumatized populations. *J Trauma Stress*. 2006;19(6):757-769.
37. Yeterian JD, Dow SJ, Kelly JH. Ensuring retention in longitudinal studies: A practical evaluation of an intensive follow-up protocol and suggested adaptations. *Internaitonal J Soc Res Metholology*. 2012;15(5):369-383.
38. Davis E, Demby H, Jenner LW, Broussard GA. Adapting an evidence-based model to retain adolescent study participants in longitudinal research. *Eval Program Plann*. 2016;54:102-111.
39. Smith LJ, McNamara PJ, King AC. Optimizing follow-up and study retention in the 21st century: Advances from teh front line in alcohol and tobacco reaerch. *Drug Alcohol Depend*. 2017;175:171-178.
40. www.phenxtooklit.org/covid19 retrieved on June 2, 2020.
41. Cohen S, Kamarck T, Mermelstein R. A global measure of perceived stress. *J Health Soc Behav*. 1983;24:386-396.
42. Hughes ME, Waite LJ, Hawkley LC, Cacioppo JT. A short scale for measuring loneliness in large surveys: Results from two population-based studies. *Res Aging*. 2004;26(6):655-672.
43. Booth ML. Assessment of physical activity: An international perspective. *Res Q Exerc Sport*. 2000;71(2):s114-120.
44. Hahn EA, DeVellis RF, Bode RK, et al. Measuring social health in the patient-reported outcomes measurement information system (PROMIS): Item bank development and testing. *Qual Life Res*. 2010;19(7):1035-1044.
45. Schaufeli WB, Bakker AB, Salanova M. The measurement of work engagement with a short questionnaire: A cross-national study. *Educ Psychol Meas*. 2006;66(4):701-716.
46. Endicott J, Nee J. Endicott work productivity scale (EWPS): A new measure to assess treatment effects. *Psychopharmacol Bull*. 1997;33(1):13-16.
47. Straus MA, Douglas EM. A short form of the Revised Conflict Tactics Scales, and typologies for severity and mutuality. *Violence Vict*. 2004;19(5):507-520.
48. Funk JL, Rogge RD. Testing the ruler with item response theory: Increasing precision of measurement for relationship satisfaction with the Couples Satisfaction Index. *Relationship Satisfac.* 2015; 21(October):572-583.
49. Grasso DJ, Briggs-Gowan MJ, Ford JD, Carter AS. *The Epidemic – Pandemic Impacts Inventory (EPII).* 2020.
50. Harris P. *All of Us Research Program COvid-19 Participant Experience (COPE) Survey (PPI)*. 2020.
